# Relationship of left piriform cortex network centrality with temporal lobe epilepsy duration and drug resistance

**DOI:** 10.1111/ene.70018

**Published:** 2025-02-13

**Authors:** Felix Zahnert, Paul Reichert, Louise Linka, Lars Timmermann, André Kemmling, Alexander Grote, Christopher Nimsky, Katja Menzler, Marcus Belke, Susanne Knake

**Affiliations:** ^1^ Epilepsy Center Hesse, Department for Neurology University Hospital Marburg, Philipps University Marburg Marburg Germany; ^2^ Department for Neuroradiology University Hospital Marburg, Philipps University Marburg Marburg Germany; ^3^ Department for Neurosurgery University Hospital Marburg, Philipps University Marburg Marburg Germany; ^4^ Center for Mind, Brain and Behavior (CMBB) Philipps‐University Marburg Marburg Germany; ^5^ LOEWE Center for Personalized Translational Epilepsy Research (Cepter) Goethe‐University Frankfurt Frankfurt Germany; ^6^ Core Facility Brainimaging, Faculty of Medicine University of Marburg Marburg Germany

**Keywords:** anti‐seizure medication, connectome, diffusion MRI, epilepsy surgery, graph theory

## Abstract

**Background:**

We investigated the relationship of piriform cortex (PC) structural network centrality with drug resistance and epilepsy duration as markers of sustained epileptic activity.

**Methods:**

PCs were manually delineated on retrospectively collected 3D‐T1‐MRI images of patients with temporal lobe epilepsy (TLE). Connectomes were computed from diffusion MRI scans, including the PC as network nodes. Betweenness centrality (BC) and node degree were computed and compared across drug‐resistant versus drug‐sensitive patients. Correlations of centrality metrics with the duration of epilepsy were calculated.

**Results:**

Sixty‐two patients (36 females, 43/62 drug‐resistant) were included in the main analysis. Greater centrality of the left PC was associated with drug resistance (degree: *p* = 0.00696, *d* = 0.85; *BC*: *p* = 0.00859, *d* = 0.59; alpha = 0.0125). Furthermore, left PC centrality was correlated with epilepsy duration (degree: rho = 0.39, *p* = 0.00181; *BC*: rho = 0.35, *p* = 0.0047; alpha = 0.0125). Results were robust to analysis of different parcellation schemes. Exploratory whole‐network analysis yielded the largest effects in the left PC. Finer parcellations showed stronger effects for both analyses in the left olfactory cortex rostral to PC. In 28 subjects who had received epilepsy surgery, a trend of smaller centrality in patients with ILAE I outcome was observed in this area.

**Conclusions:**

We demonstrated an increased centrality of the left PC in patients with drug‐resistant TLE, which was also associated with the epilepsy duration. Recurring seizures over long periods may lead to changes of network properties of the PC. Large effects immediately rostral to our delineated PC region suggest a role of olfactory cortex anterior to the limen insulae in epileptogenic networks.

## INTRODUCTION

The piriform cortex (PC) is a region of three layered primary olfactory cortex located at the junction of the frontal and temporal lobes [1]. In health, it is associated with odor quality coding [2], encoding of valence information [3] and further functions such as memory [4]. The PC is connected to a wide range of olfactory, limbic and distant regions [5, 6, 7], and there is evidence that this connectivity may be asymmetric [8].

The PC has been shown to be highly susceptible to kindling and to chemical epileptogenesis in rodents [9, 10, 11]. Induced status epilepticus in rodents results in the loss of GABAergic interneurons of this region [9]. Stimulation of its anterior portion can lead to spontaneous generation of seizures which propagate to posterior PC and to distant loci in rodents [11]. In humans, several EEG‐functional MRI experiments showed PC activations during interictal epileptiform discharges, independent of the irritative zone [12, 13]. Decreased volumes within the ipsilateral PC have been found to correlate with the duration of epilepsy [14] and to predict worse seizure control after an anterior temporal lobe resection (ATLR) [15]. A larger extent of resection within the PC during ATLR has been associated with better seizure control [15], and this finding has been reproduced by independent groups [16, 17, 18, 19]. A stronger integration of the PC in epileptogenic networks with the assumption of a central role here has been proposed as a mechanism for both its involvement in epileptic activity in various epilepsies and for the necessity of its removal to achieve seizure freedom in many cases [15, 20].

However, studies on the network properties of the PC in human epilepsies are scarce. An fMRI‐based multivariate pattern analysis study found stronger degree centrality, a measure of “hubness” of a brain region, in PC voxels to be predictive for the classification of patients with epilepsy compared to healthy controls [21]. The structural connectivity of the human PC has not been analyzed in patients with epilepsy, although diffusion MRI data are frequently acquired in the clinical setting.

Based on its proposed involvement in epileptogenic networks, its susceptibility to epileptogenic stimulation in rodents, the effects of its removal on surgical outcomes and its loss of volume with the duration of epilepsy, we hypothesized that the PC would exhibit an increased importance in neural networks of patients with continued exposure to epileptic activity compared to those with adequate seizure control.

We computed structural connectomes in patients with temporal lobe epilepsy (TLE) and analyzed the relationship between parameters of network centrality within the PC with the response to anti‐seizure medication and the duration of epilepsy.

## METHODS

### Study population

Patients were screened and included retrospectively at the Epilepsy‐Center Hesse of the University Hospital Marburg. Inclusion criteria were (i) the diagnosis of TLE based on electroclinical features and (ii) availability of a diffusion MRI scan suitable for tractography. Clinical diagnoses of TLE were reviewed and patients were included if the following criteria were fulfilled: (a) ictal semiology indicative of TLE [22] with (b) concordant interictal epileptiform discharges or seizures recorded on EEG confined to the temporal region, and (c) the lack of extratemporal structural lesions on MRI. Patients were excluded if semiological features indicated potential extratemporal epileptogenic zones.

Drug‐resistance was defined as continued recurring seizures after ≥2 adequately dosed regimen of ASM, either in monotherapy or in combination. Subjects were included in the drug‐sensitive cohort if they (i) did not meet criteria for drug‐resistance and (ii) if they were seizure free for at least three inter‐seizure intervals at last follow‐up on ASM after ≤2 ASM treatments. Of 64 patients with TLE who had adequate MRI data and clinical data available, two subjects were excluded from the analysis of drug‐resistance as neither criteria for drug‐resistance, nor criteria for the lack thereof were fulfilled.

#### Standard protocol approvals, registrations, and patient consents

The study was approved by the local IRB (IRB00011440), which did not require dedicated collection of consent for this retrospective data analysis.

### MRI acquisition

All scans were acquired on a 3T‐Siemens Trio scanner (Siemens Healthcare, Erlangen, Germany). If multiple scans were available in one subject, the latest scan with the best available quality was used.

3D‐T1‐weighted scans (MPRAGE) were acquired with three different protocols. Sixty‐three images were acquired with 1 mm isovoxel resolution, TR = 1.9 ms, TI = 0.9 s, Flip angle = 9°, TE = 2.26 and 2.52 ms (*n* = 18 and 45 subjects, respectively), and one with 0.8 mm isotropic, TR = 1.9 ms, TI = 0.9 s, Flip angle = 9°, TE = 2.64 ms. Echo Planar diffusion MRI scans were acquired with five different protocols with resolutions ranging from 1.3 mm isotropic to 2 × 2 × 2.4 mm. Gradient directions ranged from 30 to 80, acquired b0‐images ranged from 1 to 5 and *b*‐values ranged from 1000 to 2000. The most common protocol (*n* = 51) had 30 gradient directions, a single *b*‐value = 1000 and one b0 image (TE = 104 ms, TR = 10.7 s, Flip angle = 90°). Diffusion MRI protocols are listed in detail in the Supplementary Material (Table S1).

### MRI processing

#### Structural MRI

Individual T1‐scans were processed using the recon‐all pipeline as available in freesurfer [23] (https://surfer.nmr.mgh.harvard.edu/) for B0‐correction, brain extraction, surface extraction and individual parcellation and segmentation of cortical and subcortical areas.

Bilateral PCs were manually delineated on structural images in native space according to an established protocol described in detail elsewhere [15]. Delineation of the PC was conducted in the AP direction on coronal sections of the T1‐scan and was started at the limen insulae. Posteriorly, the PC decreased in its frontobasal extent and was further extended into superficial, mesial parts of the amygdala. Delineation was stopped with the emergence of the mammillary bodies posteriorly. Delineation was conducted by an investigator (FZ) who was blinded for clinical outcomes of the respective patients. Image resolutions in this study deviated from the 1.1 mm isotropic resolution used in the original publication of the utilized protocol for PC delineation [15]. As this protocol was not designed on a strict slice‐by‐slice basis, adherence to the landmark‐based delineations proposed therein was still possible.

The Desikan parcellation [24] was used for the main analysis. Supratentorial subcortical gray matter was included in the analysis, and including bilateral PC, 82 regions resulted for network construction. The parcellation and PC‐ROI were registered from individual structural space to the diffusion image via boundary‐based registration (freesurfer bbregister [25]). It was ensured that no parcel overlapped with the Piriform‐ROI by deleting overlapping voxels from these parcels. Furthermore, only voxels at the gray/white‐matter interface were kept for later tractography.

#### Diffusion MRI

Preprocessing of diffusion scans included brain extraction and correction for eddy currents (FSL eddy [26]). Orientations of diffusion were estimated using the GPU version of the FSL tool BedpostX [27]. Probabilistic tractography was conducted using the GPU version of FSL probtrackX [28] in network mode, seeding 5000 streamlines per voxel of each generated parcel. An exclusion mask was applied to cortical gray matter outside the gray/white interface to avoid spurious connections across adjacent gyri. Cerebrospinal fluid, ventricles, and infratentorial structures were excluded from tracking, and the entire white matter was used as a waypoint mask.

To analyze the robustness of our results, networks were also generated using the Destrieux parcellation [29], with 164 cortical and subcortical ROI, as well as the 200‐region Schaefer functional parcellation [30], containing 214 ROI including subcortical regions. The latter was registered from standard MNI152 space to individual diffusion space. Processing of parcels was the same as reported for the main analysis above.

#### Processing of network matrices

Tractography resulted in individual asymmetric matrices of 82 × 82 ROIxROI connections, with each element containing a streamline count as a measure of connection strength. Processing was conducted analogously to procedures published elsewhere [31]. Matrices were (i) normalized region‐wise for the total streamline count (waytotal), (ii) rendered symmetric and (iii) thresholded, as probabilistic tractography commonly leads to spurious connections [32], while brain networks are physiologically not fully connected, but can be rather sparse [33, 34, 35]. To minimize bias introduced by network thresholding and to avoid multiple comparisons problems introduced by interrogation of multiple network thresholds, individual matrices were thresholded at a range of 20%–40% network density (step size 1%) for later integration of network metrics across densities [33, 36]. The lower boundary of 20% was chosen as we found networks to fragment unphysiologically in a small fraction of healthy subjects at densities as high as 15% in a previous study [31].

#### Graph theoretic metrics

At each network density, graph theoretic measures of centrality, or “hubness,” were computed for bilateral PC, as well as for the remaining regions for later explorative analysis. Node degree and betweenness centrality (BC) were calculated for each region using functions from the brain connectivity toolbox [37] (https://sites.google.com/site/bctnet/). Node degree is an intuitive metric which indicates the number of connections a parcel has with the rest of the network, while BC measures the number of shortest paths within a network that traverse a region. In patients with TLE undergoing epilepsy surgery, increased BC in the temporal lobe has been shown to be a potential indicator of ongoing postoperative seizures [38].

**FIGURE 1:**
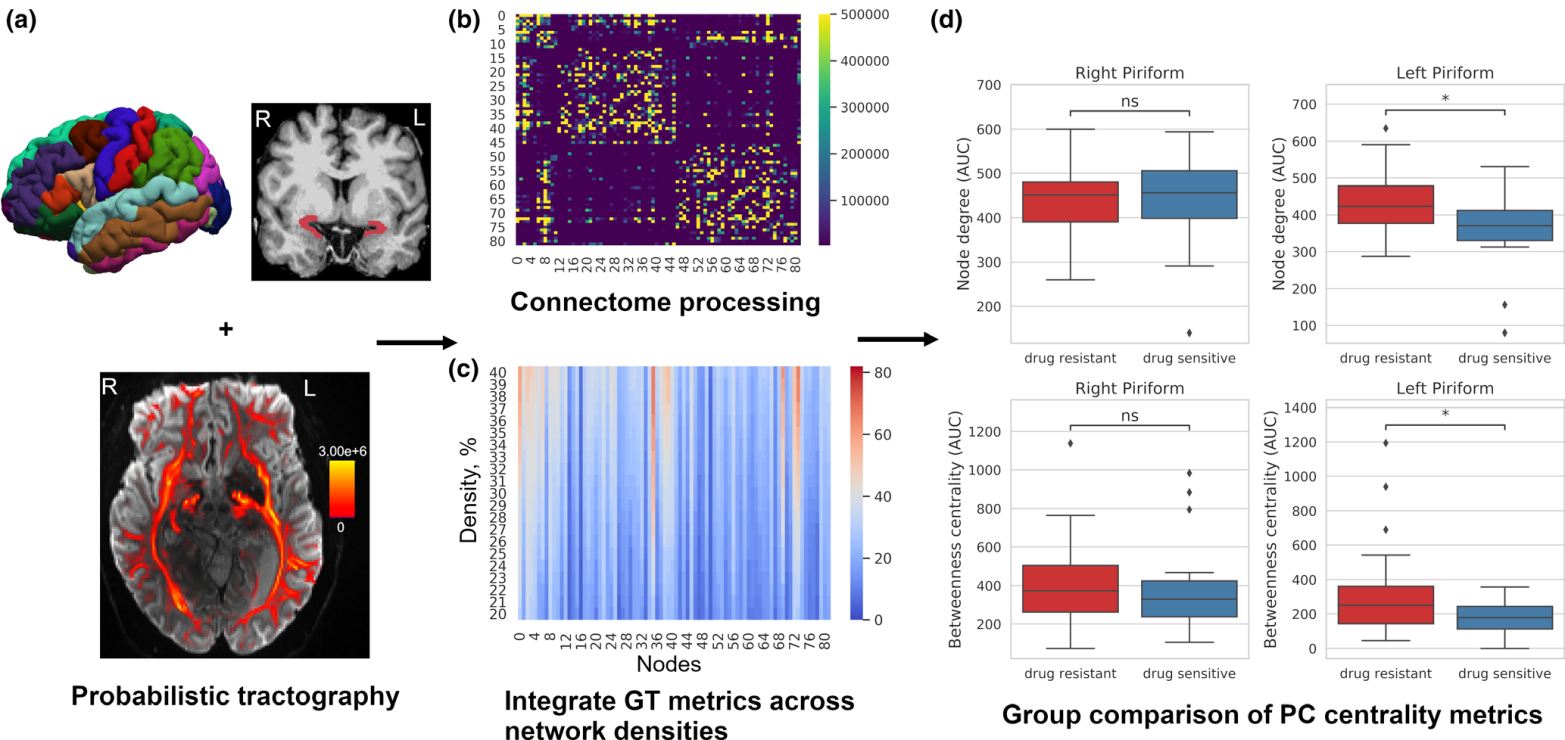
Overview of study methodology. (a) Individual piriform cortices were delineated manually and added to a cortical and subcortical parcellation of the brain (Desikan, Destrieux2009, and Schaefer2018 parcellations analyzed; the exemplary Desikan parcellation is depicted here on the pial surface). The bottom image depicts voxelwise streamline visitation counts overlaid on the mean diffusion weighted image of one subject. Streamlines were seeded from all voxels at the gray–white boundary within each volume‐space parcel and a ROI × ROI connectivity matrix was constructed. (b) Raw single‐subject 82 × 82 adjacency matrix, color‐coded (and thresholded between 1 k and 500 k streamlines visualization) for shared streamline counts between parcels. Resulting adjacency matrices were normalized, rendered symmetric and thresholded at network densities between 20% and 40%. (c) Graph theoretic metrics were computed at each network density and for each region, resulting in the depicted exemplary single‐subject matrix with 21 (densities) × 82 (regions) elements; node degree is color coded. Graph theoretic metrics were then integrated across thresholds to obtain one value per node. (d) Hypothesis testing of differences in PC network integration in patients with drug resistance versus patients who were drug‐sensitive. * = *p* < 0.05, corrected.

The obtained graph theoretic metrics were then summed across network densities to obtain one measure per patient (i.e., an area under the curve of all centrality values for the range of 20%–40% connection densities) [39]. We are going to continue to refer to these integrated metrics as “node degree” and “betweenness centrality” (BC) for better legibility (Figure 1).

### Statistical analysis

Centrality metrics of bilateral PC were compared across the groups of drug‐resistant versus drug‐responsive TLE via permutation tests with 100.000 permutations. The alpha was set to 0.0125 as four tests were conducted (bilateral PC for both metrics). The same analysis was repeated for the left PC for confirmation of results in both the Destrieux‐ and Schaefer‐networks.

Adjustment for clinical covariates was conducted using ordinary least squares linear regression (https://www.statsmodels.org/stable/index.html). The covariate “MRI lesion” was a dummy variable constructed from cases with a lesional MRI or hippocampal sclerosis (0) versus patients with other lesions (1).

Furthermore, Spearman correlations (r_s_) were computed between the centrality metrics within the PC and the duration of epilepsy, and again, the alpha was set to 0.0125.

Afterward, exploratory multiple univariate testing of centrality metrics was conducted for both the comparison of drug‐resistant versus responsive TLE as well as their correlations with the duration of epilepsy. Here, *p*‐values were FDR corrected for 82 comparisons, corresponding to 82 regions tested (164 or 214 comparisons for the Destrieux‐ and Schaefer parcellations, respectively).

After obtaining associations of PC centrality with drug‐resistance, we performed an exploratory follow‐up analysis in a subsample of patients who had received epilepsy surgery. Here, we hypothesized that increased centrality of left olfactory regions, which was associated with drug‐resistance and epilepsy duration, may also be associated with poor seizure control after epilepsy surgery. Seizure freedom was defined here as an ILAE‐1 outcome after a postoperative follow‐up of at least 1 year. A permutation *t*‐test with 100.000 permutations was conducted. The alpha was set to 0.005 to conservatively correct for 10 comparisons.

### Visualization

Glass brains were visualized using the python package nilearn (https://nilearn.github.io/stable/index.html). Visualization of individual parcels on the MNI152 brain was achieved using the freesurfer image viewer freeview. The python package seaborn was used to plot box plots and heat maps [40].

## RESULTS

### Clinical characteristics

Sixty‐four patients with TLE (59.7% females) were included in the study. Sixty‐two patients had adequate available information on their ASM response and were included in the main analysis. Table 1 highlights clinical characteristics of this study population. Two subjects had only information on their response to epilepsy surgery available and were therefore included in the auxiliary analysis of surgical outcomes only.

**TABLE 1:** Clinical characteristics of the study population with information on drug resistance available (*n* = 62). “LEAT” (Long‐term epilepsy‐associated tumors) as an epileptogenic lesion included dysembryoplastic neuroepithelial tumors and Gangliogliomas.

Characteristics	Overall, *n* (%)	Drug resistant, *n* (%)	Drug responsive, *n* (%)
Sex			
Male	26 (41.9)	17 (39.5)	9 (47.4)
Female	36 (58.1)	26 (60.5)	10 (52.6)
Laterality of TLE			
Left	37 (59.7)	25 (58.1)	12 (63.2)
Right	20 (32.2)	13 (30.2)	7 (36.8)
Bilateral	4 (6.5)	4 (9.3)	0 (0)
Unclear	1 (1.6)	1 (2.3)	0 (0)
Drug resistance	43 (69.4)		

Characteristics	Overall, M (SD)	Drug resistant, M (SD)	Drug responsive, M (SD)
Age of onset in years	23.7 (16.5)	21.1 (16)	29.5 (16.7)
Duration of epilepsy at *t*(MRI) in years	14.5 (13.1)	18.9 (13.4)	4.7 (4)

Characteristics	Overall, *n* (%)	Drug resistant, *n* (%)	Drug responsive, *n* (%)
Etiology			
Structural	38 (61.3)	27 (62.8)	11 (57.9)
Unclear	24 (38.7)	16 (37.2)	8 (42.1)
Epileptogenic lesion on MRI			
Hippocampal sclerosis	26 (41.9)	20 (46.5)	6 (31.6)
Cavernoma	4 (6.5)	2 (4.7)	2 (10.5)
LEAT	5 (8.1)	2 (4.7)	3 (15.8)
Focal cortical dysplasia	3 (4.8)	3 (7)	–
No lesion	24 (38.7)	16 (37.2)	8 (42.1)
Occurrence of BTCS (*n* = 61)	41 (67.2)	32 (74.4)	9 (50)
Mean clinical follow‐up of seizure freedom on ASM in months (min‐max)	–	–	23 (4–72)
Overall number of ASM tried (mean, min‐max)	–	6.3 (2–14)	1.37 (1–2)

### Network centrality of the piriform cortex

#### Drug resistance

**FIGURE 2:**
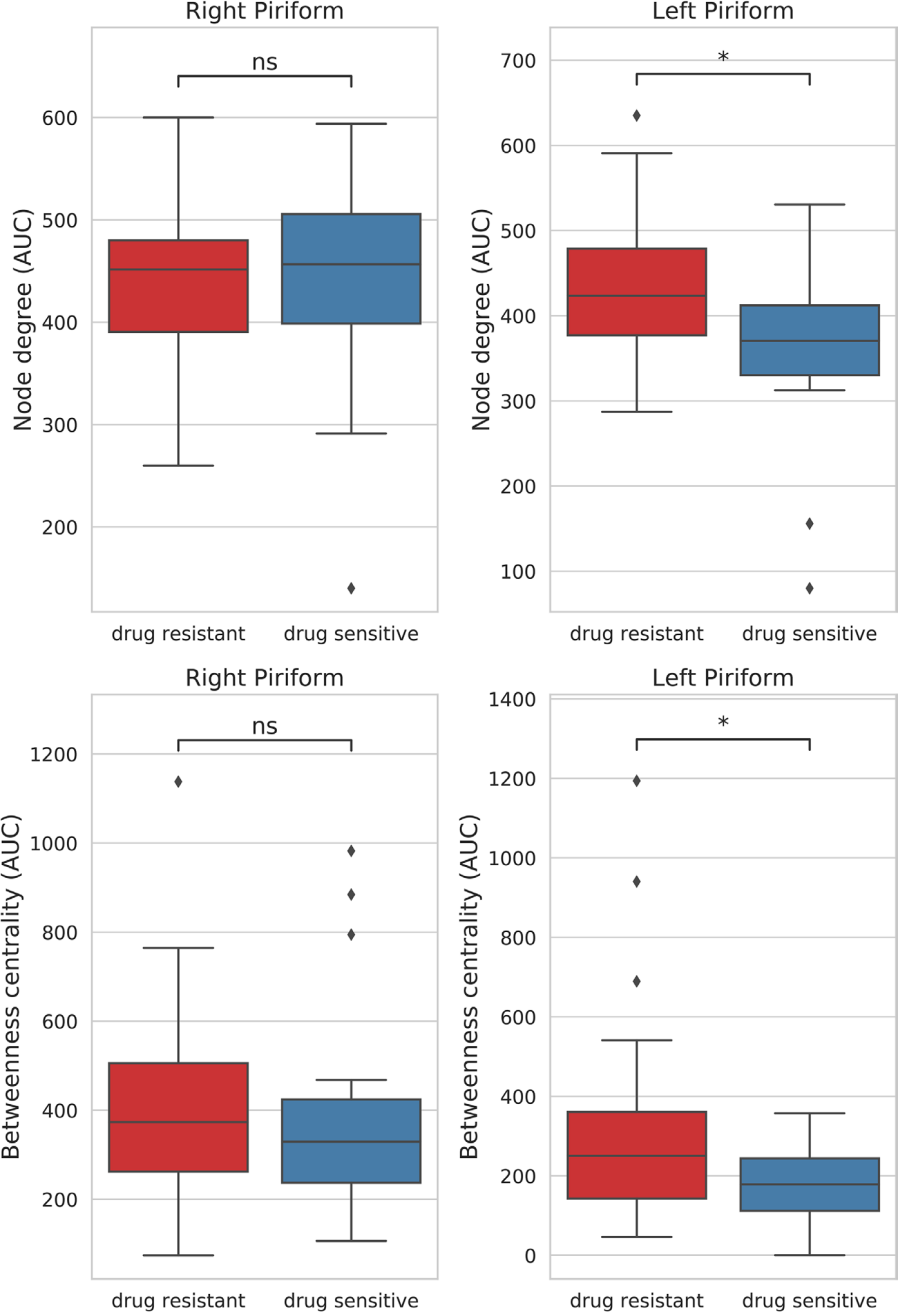
Graph theoretic metrics (*y*‐axis) are depicted in dependence of the response to anti‐seizure medications (*x*‐axis). The first row shows results for node degree, betweenness centrality is shown below. * = *p* < 0.05, Bonferroni corrected for four comparisons. ns, not significant.

The left PC showed significantly stronger network centrality metrics in subjects with drug‐resistant TLE compared to those who were not drug‐resistant (degree: *t* = 2.72, *p* = 0.00696, *d* = 0.85; *BC*: *t* = 2.74, *p* = 0.00859, *d* = 0.59; two‐tailed, alpha = 0.0125). In the right PC, no significant effect was observed (Figure 2).

Drug‐resistance remained associated with an increased node degree in the left PC after adjustment for the duration of epilepsy using linear regression (*t* = 2.29, *p* = 0.026; Model adj. *R* ^2^ = 0.12, df = 2, F‐statistic = 4.95, *p* (F‐statistic) = 0.0103). This association remained significant after additional adjustment for sex, laterality of epilepsy, type of lesion on MRI imaging and the age of epilepsy onset as covariates (*t* = 2.12, *p* = 0.038; Model adj. *R* ^2^ = 0.11, df = 5, F‐statistic = 2.51, *p* (F‐statistic) = 0.0405). Associations of increased BC of the left PC with drug‐resistance did not remain significant after adjustment for these covariates.

The effect within the left PC was then confirmed in networks generated from two further parcellation schemes (Table 2). Here, however, lower effect sizes were detected (*Destrieux‐parcellation*, 164 nodes: degree: *t* = 2.05, *p* = 0.045, *d* = 0.61; *BC*: *t* = 2.35, *p* = 0.0234, *d* = 0.5; *Schaefer 2018 parcellation*, 214 nodes: degree: *t* = 2.05, *p* = 0.0427, *d* = 0.52; *BC*: *t* = 1.48, *p* = 0.0736, *d* = 0.28; all *p*‐values uncorrected).

**TABLE 2:** Depicted are statistics for the top three regions from the Destrieux and Schaefer parcellations for the test “drug resistant versus not drug resistant,” sorted by ascending *p*‐values. “Lh_S_orbital_med_olfact” and “17Networks_LH_LimbicB_OFC_1” are both left caudal orbitofrontal parcels in close vicinity to our PC‐ROI. “Lh_S_orbital‐H_Shaped” and 17Networks_RH_LimbicB_OFC_2 are part of the left and right ventrolateral orbitofrontal cortex. “Rh_S_oc_middle_and_lunatus” is a part of lateral occipital cortex and “17Networks_RH_SalVentAttnA_ParMed_1” is a small parcel situated in the cingulate cortex.

Parcellation	Region	*t*‐stat	*p* (corrected for n nodes)	Cohen's *d*
Destrieux, 164 nodes				
Degree	Rh_G_subcallosal	2.26	0.03 (0.97)	0.69
	Rh_S_oc_middle_and_lunatus	2.23	0.033 (0.97)	0.61
	Lh_S_orbital_med_olfact	2.08	0.045 (0.97)	0.52
Betweenness centrality	Lh_S_orbital_med_olfact	3.99	0.00066 (0.107)	0.87
	Lh_S_orbital‐H_Shaped	2.45	0.0208 (0.98)	0.59
	Lh_piriform	2.35	0.0234 (0.98)	0.51
Schaefer 2018, 214 nodes				
Degree	17Networks_LH_VisCent_ExStr_4	3.25	0.00262 (0.56)	0.87
	17Networks_LH_LimbicB_OFC_1	2.91	0.00624 (0.6)	0.71
	17Networks_RH_LimbicB_OFC_2	2.79	0.00838 (0.6)	0.73
Betweenness centrality	17Networks_LH_LimbicB_OFC_1	2.67	0.00158 (0.28)	0.5
	17Networks_RH_SalVentAttnA_ParMed_1	3.07	0.00266 (0.68)	0.66
	17Networks_LH_VisCent_ExStr_4	2.81	0.01021 (0.73)	0.64

An exploratory analysis of effect sizes within the PC for patients with left and right TLE separately indicated larger effects in the left PC in patients with right TLE (Table S2). Again, no relevant effects were detected in the right PC.

#### Correlation with the duration of epilepsy

We then analyzed whether centrality measures of the PC were correlated with the duration of epilepsy (information available in 61/62 subjects). A significant correlation was observed again in the left PC (degree: r_s_ = 0.39, *p* = 0.00181; *BC*: r_s_ = 0.35, *p* = 0.0047; alpha = 0.0125), while no significant correlation was apparent in the right PC.

To investigate whether age at MRI acquisition contributed to this finding, Spearman correlations of centrality metrics in the left PC with the age at the time of MRI acquisition were computed. No correlations were observed for either metric (degree: r_s_ = 0.039, *p* = 0.77; *BC*: r_s_ = 0.06, *p* = 0.65).

These results were confirmed within networks based on both the Schaefer2018 and the Desikan parcellation (Table S3).

### Exploratory analysis of the entire network

#### Whole network: drug resistance

**FIGURE 3:**
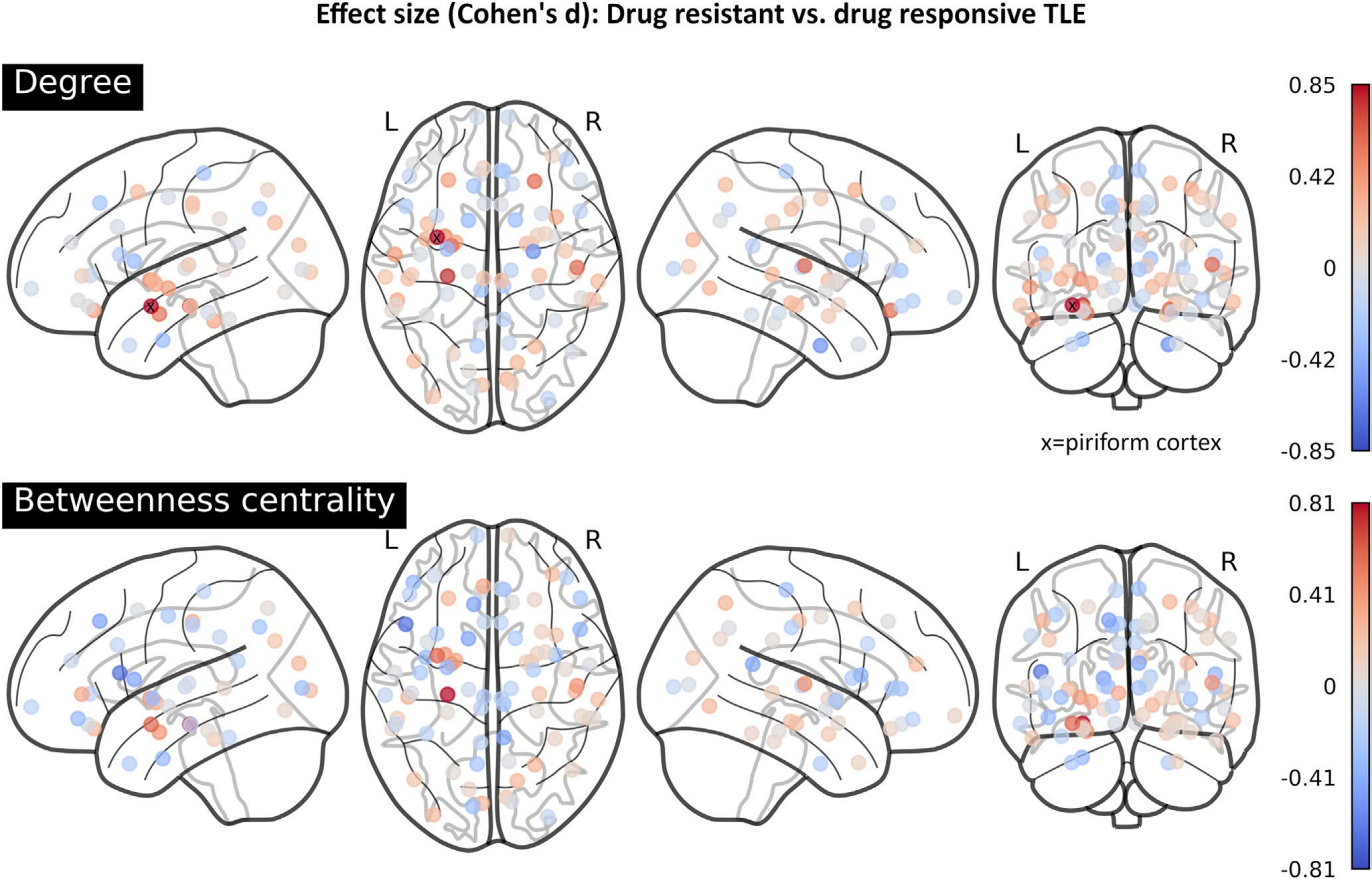
Effect sizes of multiple univariate testing for differences in node degree (above) and betweenness centrality (below) across groups of drug responsive versus drug‐resistant TLE. Nodes are depicted according to the Desikan parcellation, including additional subcortical ROI. The largest positive effects were observed in the left mesial temporal lobe, that is, in the left PC (degree: d = 0.85, betweenness centrality: d = 0.59) and the left hippocampus (degree: d = 0.78, betweenness centrality: d = 0.81).

After statistical testing of metrics within the PC, exploration of effect sizes across the entire network was conducted. The left PC showed the strongest effect of increased degree in drug‐resistant subjects across the entire network, while for BC, it had the second strongest effect after the left hippocampus (Figure 3).

##### Drug resistance: Destrieux and Schaefer parcellations

In the finer‐grained Destrieux‐ and Schaefer‐parcellations, strong effects were observed in orbitofrontal regions close to PC (Table 2 and Tables S6–S9). In some areas, these orbitofrontal parcels overlapped with the PC's anterior aspects according to an alternative definition of PC (Mai et al. 2015). No region withstood FDR‐correction for respective 164 and 214 comparisons.

#### Whole network: correlation with the duration of epilepsy

**FIGURE 4:**
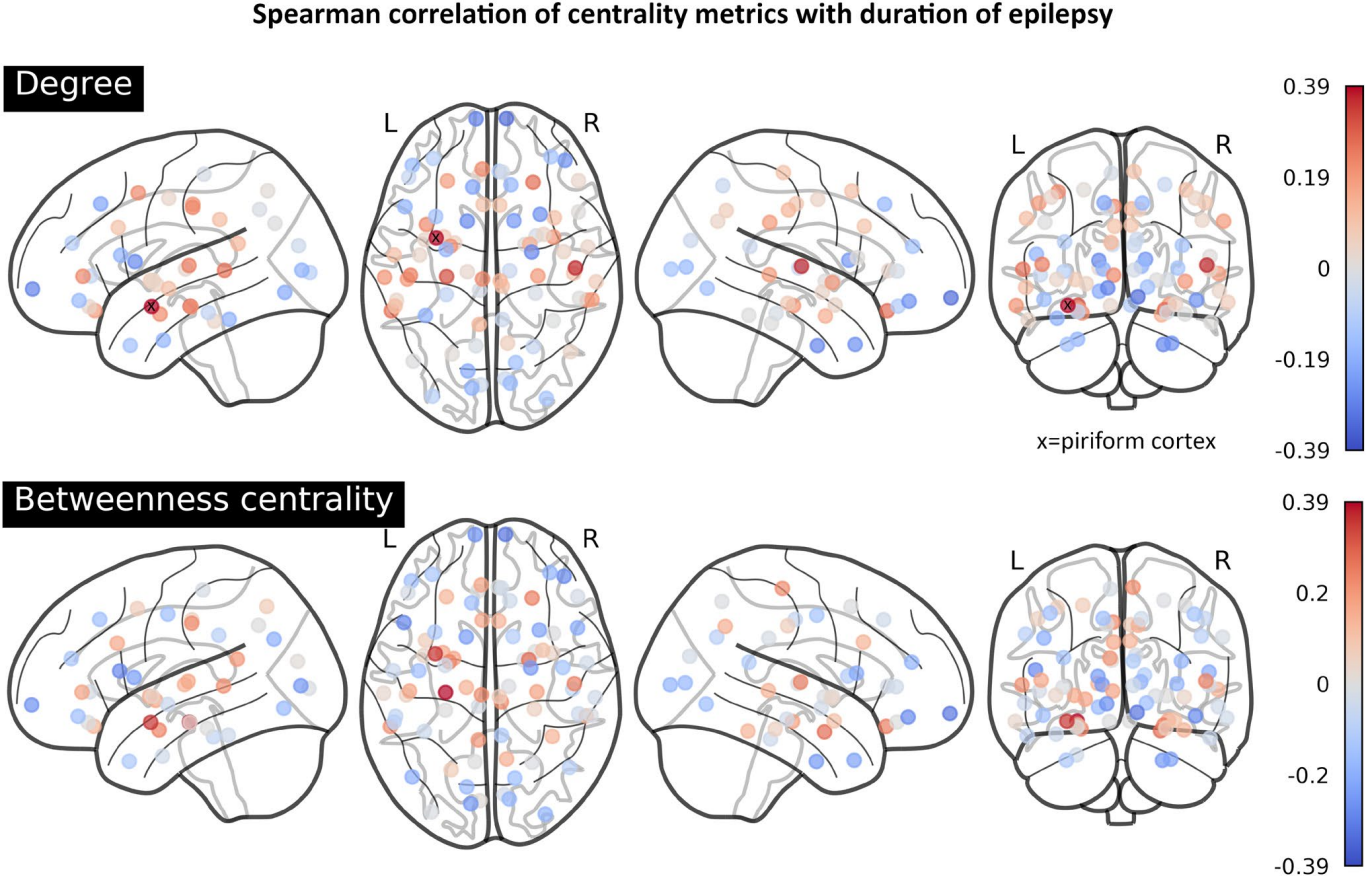
Spearman correlations of centrality metrics with the duration of epilepsy across the entire network. The largest correlations were observed in the left piriform cortex (degree: R_s_ = 0.39, betweenness centrality: R_s_ = 0.35) and the left hippocampus (degree: R_s_ = 0.31, betweenness centrality: R_s_ = 0.39). The right transverse temporal gyri furthermore showed comparatively large correlations (degree: R_s_ = 0.35).

The strongest correlations with the duration of epilepsy were also observed in the centrality of the left PC and hippocampus (Figure 4). No correlation survived FDR‐correction for 82 comparisons.

##### Correlation with the duration of epilepsy: Destrieux and Schaefer parcellations

Within the Schaefer parcellation, a significant, moderate correlation was observed for a left caudal orbitofrontal ROI in close vicinity of the PC (visualized in Figure S1), surviving correction for 214 comparisons (17Networks_LH_LimbicB_OFC_1; degree: *r* = 0.44, *p* = 0.034; *BC*: *r* = 0.5, *p* = 0.0073; *p*‐values FDR corrected for 214 comparisons). This ROI overlaps with the Lh_S_orbital_med_olfact ROI from the Destrieux parcellation (Table 2), which has shown a strong effect of greater BC in drug‐resistant TLE.

Furthermore, a significant anti‐correlation of node degree with the duration of epilepsy was observed within a parcel substantially overlapping with the pars opercularis of the left inferior frontal gyrus. (LH_DefaultB_PFCv4; *r* = −0.45, *p* = 0.034, corrected for 214 comparisons).

Further results from these additional parcellations are reported in the Supplementary Material—Data S1.

### Centrality of left olfactory cortex and seizure control after epilepsy surgery

After demonstrating increased centrality of the left PC and adjacent olfactory cortex in patients with drug‐resistance and longer epilepsy duration, we explored if this preoperative increase in centrality may also relate to outcomes after epilepsy surgery.

Clinical characteristics of this sample of 28 operated patients (26 patients from the above analysis and two additional patients without data on ASM response) are described in Table S16.

Based on the above results, three regions of interest were analyzed: the left PC (all three parcellations), the 17Networks_LH_LimbicB_OFC_1 region from the Schaefer2018 parcellation (cf. Figure S1) and the Lh_S_orbital_med_olfact region from the Destrieux parcellation.

Moderate‐to‐strong effects of smaller centrality in seizure free patients (ILAE 1 outcome) were observed only in the 17Networks_LH_LimbicB_OFC_1 region within the Schaefer2018 parcellation (degree: *t* = −2.51, *p* = 0.0117, *d* = −0.95; *BC*: *t* = −1.49, *p* = 0.0437, *d* = −0.57; *p*‐values uncorrected), although notwithstanding Bonferroni correction for 10 comparisons (Table S17).

## DISCUSSION

We demonstrated an increase in network centrality of the left PC in subjects with drug‐resistant TLE. Together with the concomitant association of this region's centrality with the duration of epilepsy, it is possible that ongoing seizures over long periods of time may contribute to aberrant network properties of the PC.

The PC receives extensive afferent projections from the olfactory bulb [41] and has extensive connectivity to other olfactory regions, such as reciprocal connections to the orbitofrontal cortices (OFC), or connections with limbic regions such as the cingulate (cf. Figure S9) and further cortical and subcortical areas [5, 6, 7, 42]. Previous structural and functional MRI studies demonstrated strong connectivity between the frontal aspect of the PC with bilateral basal ganglia and motor [43, 44]. Furthermore, the PC has been shown to exhibit strong structural and functional connectivity with visual areas [43]. It can be speculated that this connectivity of primary visual, olfactory, and motor cortices might support multimodal integration, potentially serving a role in food grasping [45]. Suppression of associative inputs to the PC via Baclofen intake inhibits pattern separation of distinct odors in the human PC [46]. The specific blockade of afferents to the PC may be of interest for future studies into its epileptogenicity, especially in the light of its increased centrality in drug‐resistant patients.

Both BC and node degree are markers of the hubness of a brain region, and thereby of its importance in the entire network [47]. Broad connectivity of the PC in focal epilepsy is also implied by its activation during interictal epileptiform discharges, irrespective of the epileptogenic zone [12, 13]. The structural connectome is plastic [48], and the PC is highly susceptible to kindling [10] and chemical stimulation [49]. It is therefore possible that the PC may adopt a more central role in the epileptogenic network under sustained exposure to uncontrolled epileptic activity. We speculate that this aberrant hubness of a PC embedded in an epileptogenic network may contribute to the dependence of seizure freedom after ATL on the extent of its resection. This may be corroborated by our investigation of a subsample of 28 subjects who underwent epilepsy surgery, where we observed a trend of increased hubness of left olfactory cortex in patients with recurring seizures. Betweenness centrality of temporal lobe regions has been used with some success to predict outcomes after epilepsy surgery [38]. A recent study (preprint) found decreased similarity of functional connectivity profiles between the left and right PC in drug‐resistant TLE, and patients with especially low similarity had worse postsurgical outcomes [50]. Interestingly, here, too, only the left PC showed differences in whole‐brain connectivity compared to healthy controls [50].

Based on the above prior research and on our results, we encourage the investigation of the predictive value of preoperative PC‐ and caudal orbitofrontal‐network metrics for seizure freedom after temporal lobe epilepsy surgery.

Using two alternative, finer parcellations, stronger effects were observed in olfactory regions immediately rostral to the PC ROI we had delineated. Other authors include areas rostral to the limen insulae in their definitions of PC (“anterior piriform cortex”) [51, 52]. It is therefore possible that had these anterior regions been included in our PC ROI, even stronger effect sizes might have been obtained. Animal studies have shown that kindling of the anterior PC in rats can lead to its epileptogenic transformation, with arising seizures being conducted to posterior PC and distant regions [11]. For consistency with the epileptological literature [15, 16, 53], we applied an established definition of PC in our analyses, but the anatomy of the human PC remains poorly understood, and different definitions of the PC should be tested in future studies into its epileptogenicity [43].

No effects were evident in the right PC. In healthy subjects, bilateral PCs are asymmetric in volume [14] and in their structural connectivity, with stronger connections of the left PC to the ipsilateral thalamus [8]. Both the PC and thalamus are commonly involved in epileptogenic networks [20, 51] and differences in PC connectivity could lead to preferential involvement of the left PC in such circuits. In addition, a recent study found an effect of resection volumes of the left, but not the right PC on outcomes after ATLR [52], so that a lateralized, stronger involvement of the left PC in epileptogenic networks may be possible.

### Limitations

The main limitation of this study is the rather small, retrospective cohort used for this study. The laterality of TLE was heterogenic, and statistical analyses of isolated right or left TLE were not feasible due to the small sample size. Left and right TLE seem to show distinct changes in network properties [53], and effects of increased PC centrality were larger in right TLE in the present study. Therefore, separate analysis of both entities could be worthwhile in the future. Furthermore, the etiology of TLE varied across subjects. Following this first analysis into the network properties of the PC in TLE, distinct clinical phenotypes should now be analyzed (e.g., left mTLE with hippocampal sclerosis) for potential biomarker development.

While the same scanner was used in all subjects, protocols varied. Although graph theoretic metrics are quite robust even across different scanner types [54], the analysis of uniform protocols across all subjects would have been ideal. Diffusion MRI scans had relatively low angular resolution (30 gradient directions in most cases), which, however, demonstrated that differences in PC centrality can be detected with clinical diffusion scans.

## CONCLUSION

Drug‐resistance and epilepsy duration were associated with centrality measures of the left, but not the right PC. The observed results were robust to analysis of two different parcellations, albeit with smaller effect sizes and with few other, adjacent olfactory regions showing larger effects than the PC. Overall, this study highlights that the role of the PC as a network hub may increase with lasting disease burden, which may speculatively account for previously reported associations of its removal with post‐surgical seizure freedom. Different, additional definitions of PC should be explored in future connectomic analyses of well‐phenotyped cohorts to develop potential network biomarkers for seizure freedom with ASM and epilepsy surgery.

## AUTHOR CONTRIBUTIONS

**Alexander Grote:** Writing – review and editing; data curation. **André Kemmling:** Data curation; writing – review and editing. **Christopher Nimsky:** Writing – review and editing; data curation. **Katja Menzler:** Writing – review and editing; supervision. **Susanne Knake:** Conceptualization; writing – review and editing; supervision; project administration. **Lars Timmermann:** Supervision; writing – review and editing. **Louise Linka:** Data curation; writing – review and editing. **Marcus Belke:** Writing – review and editing; supervision. **Paul Reichert:** Data curation; writing – review and editing. **Felix Zahnert:** Conceptualization; investigation; writing – original draft; visualization; writing – review and editing; formal analysis; data curation.

## FUNDING INFORMATION

Felix Zahnert was supported by a grant from the German chapter of the International League Against Epilepsy (DGfE) in the form of an “Otfrid Förster grant.” Felix Zahnert was furthermore supported by the clinician scientist program of the Philipps‐University of Marburg.

## CONFLICT OF INTEREST STATEMENT

None of the authors report conflicts of interest related to this work. **F. Zahnert** has participated in a seminar on complex epilepsies funded by UCB. Felix Zahnert was supported by a grant from the German chapter of the International League Against Epilepsy (DGfE) in the form of an “Otfrid Förster grant.” Felix Zahnert was furthermore supported by the clinician scientist program of the Philipps‐University of Marburg. **P. Reichert** reports no disclosures. **L. Linka** reports no disclosures. **L. Timmermann:** Between May 2021 and February 2024 L. Timmermann received occasional payments as a consultant for Boston Scientific (before 2022), L. Timmermann received honoraria as a speaker on symposia sponsored by Boston Scientific, AbbVIE, Novartis, Neuraxpharm, Teva, the Movement Disorders Society und DIAPLAN (before 2022). The institution of L. Timmermann, not L. Timmermann personally received funding by Boston Scientific (before 2022), the German Research Foundation, the German Ministry of Education and Research, the Otto‐Loewi‐Foundation and the Deutsche Parkinson Vereinigung. Neither L. Timmermann, nor any member of his family holds stocks, stock options, patents, or financial interests in any of the above mentioned companies or their competitors. L. Timmermann serves as the president of the German Neurological Society without any payment or any income. **A. Kemmling** is consultant for Phenox, Penumbra, Stryker. **A. Grote** reports no disclosures. **C. Nimsky** is consultant for Brainlab and received speaker honoraria by GE BK medical. **K. Menzler** received speaker honoraria/consultancy fees from UCB Pharma, Eisai, and Bial. **M. Belke** reports no disclosures. **S. Knake** received speaker's honoraria from Angellini Pharma, Bial, Eisai, Epilog, Desitin, Jazz Pharma, Kanso, Merck Serono, UCB, and Zogenix.

## ETHICS STATEMENT

We confirm that we have read the Journal's position on issues involved in ethical publication and affirm that this report is consistent with those guidelines.

## Supporting information


Appendix S1.

## Data Availability

For data protection reasons, sharing of our patient data is not possible. Code for our analyses will be made available on request.
