# Supplementary material for: Relationship of left piriform cortex network centrality with temporal lobe epilepsy duration and drug resistance
Source: Eur J Neurol. 2025 Feb 13;32(2):e70018. doi: 10.1111/ene.70018 (PMC11825592; doi:10.1111/ene.70018)
Supplement: Supplementary file 1 — Appendix S1. [file ENE-32-e70018-s001.docx]

**Relationship of left piriform cortex network centrality with temporal lobe epilepsy duration and drug resistance**

**1. Diffusion weighed imaging protocols**

| **n subjects** | **Resolution (mm)** | **TE (ms)** | **TR (s)** | **FA (degrees)** | **b0 images** | **Directions** | **b-value** | **Scanner** |
| --- | --- | --- | --- | --- | --- | --- | --- | --- |
| **51** | 2x2x2.4 | 104 | 10.7 | 90 | 1 | 30 | 1000 | Siemens 3T Trio Tim |
| **8** | 2x2x2 | 90 | 7.9 | 90 | 3 | 40 | 1000 | Siemens 3T Trio Tim |
| **2** | 2x2x2 | 101 | 8.5 | 90 | 1 | 60 | 1000, 2000 | Siemens 3T Trio Tim |
| **1** | 2x2x2 | 104 | 8.7 | 90 | 5 | 80 | 1000, 2000 | Siemens 3T Trio Tim |
| **1** | 2x2x2 | 97 | 8.3 | 90 | 5 | 80 | 1000, 1500 | Siemens 3T Trio Tim |
| **1** | 1.3 x 1.3 x 1.3 | 110 | 8.1 | 90 | 4 | 60 | 1000 | Siemens 3T Trio Tim |

**Table S1.** Diffusion weighed imaging protocols. All scans were acquired on the same scanner.

**2. Analysis of piriform cortex centrality in right and left TLE patients separately**

Here, we examine effects of the laterality of TLE on effect sizes of increased network centrality in subjects with drug resistance compared to those who were drug responsive.

In patients with left TLE, the left piriform cortex showed a moderate to small, non-significant effect (degree: d=0.66, BC: d=0.39) with a trend towards greater centrality in the drug resistant subgroup (Table S2). In subjects with right TLE, however, this effect was large (degree: d=1.07, BC: d=1.01) and significant for BC, although not withstanding correction for multiple comparisons in this small subsample.

The right piriform cortex showed no association with drug resistance.

**Table S2**. Association of PC centrality with drug resistance in subjects with left and right TLE tested separately. Centrality metrics were obtained from the Desikan parcellation, as used for the main analysis.

| **Left TLE** |  |  |  |
| --- | --- | --- | --- |
|  | **t** | **P (uncorrected)** | **d** |
| *Degree* |  |  |  |
| - left PC | 2.01 | 0.0454 | 0.74 |
| - right PC | 0.83 | 0.41 | 0.31 |
|  |  |  |  |
| *Betweenness centrality* |  |  |  |
| - left PC | 1.33 | 0.199 | 0.39 |
| - right PC | 0.69 | 0.51 | 0.24 |
|  |  |  |  |
| **Right TLE** |  |  |  |
|  | **t** | **P (uncorrected)** | **d** |
| *Degree* |  |  |  |
| - left PC | 1.9 | 0.057 | 1.09 |
| - right PC | -0.04 | 0.97 | -0.02 |
|  |  |  |  |
| *Betweenness centrality* |  |  |  |
| - left PC | 2.42 | 0.0218 | 1.03 |
| - right PC | -0.33 | 0.74 | -0.18 |

**2. Correlations of left PC centrality with duration of epilepsy: Schaefer and Destrieux networks**

**Table S3**. Spearman correlations between left PC centrality metrics and the duration of epilepsy. Depicted p-values are uncorrected, the alpha was set to 0.0125

| **Parcellation** | **r_s_** | **p** |
| --- | --- | --- |
|  |  |  |
| **Destrieux**, 164 nodes |  |  |
| *degree* | 0.3 | 0.016 |
| *betweenness centrality* | 0.36 | **0.004** |
|  |  |  |
| **Schaefer** **2018**, 214 nodes |  |  |
| *degree* | 0.3 | 0.018 |
| *betweenness centrality* | 0.31 | **0.0121** |

**3. Whole network analysis: drug resistance vs. drug responsiveness**

**Table S4.** The top ten largest effects within the Desikan parcellation for node degree are highlighted. “Corr_p” = p value corrected for 82 comparisons

| Region | t_stat | p | d | corr_p |
| --- | --- | --- | --- | --- |
|  |  |  |  |  |
| Left-Hippocampus | 2.91 | 0.00632 | 0.78 | 0.29 |
| lh_piriform | 2.72 | 0.00696 | 0.85 | 0.29 |
| ctx-rh-transversetemporal | 2.26 | 0.03022 | 0.59 | 0.84 |
| ctx-rh-entorhinal | -2.04 | 0.05071 | -0.56 | 0.9 |
| Left-Amygdala | 2 | 0.0542 | 0.57 | 0.9 |
| ctx-rh-lateralorbitofrontal | 1.82 | 0.07801 | 0.59 | 0.99 |
| Left-Putamen | 1.63 | 0.10745 | 0.49 | 0.99 |
| Left-Caudate | -1.45 | 0.15663 | -0.35 | 0.99 |
| ctx-lh-superiortemporal | 1.37 | 0.18161 | 0.4 | 0.99 |
| ctx-lh-lateralorbitofrontal | 1.34 | 0.18909 | 0.39 | 0.99 |

**Table S5.** Top ten largest effects within the Desikan parcellation for betweenness centrality. “Corr_p” = p value corrected for 82 comparisons

| Region | t_stat | p | d | corr_p |
| --- | --- | --- | --- | --- |
|  |  |  |  |  |
| Left-Hippocampus | 3.29 | 0.00289 | 0.81 | 0.24 |
| lh_piriform | 2.74 | 0.00859 | 0.59 | 0.36 |
| ctx-lh-parsopercularis | -1.87 | 0.07078 | -0.67 | 0.96 |
| ctx-rh-transversetemporal | 1.83 | 0.08054 | 0.42 | 0. 96 |
| Left-Putamen | 1.77 | 0.08609 | 0.41 | 0. 96 |
| Left-Amygdala | 1.7 | 0.10341 | 0.43 | 0. 96 |
| ctx-lh-superiorfrontal | -1.58 | 0.12395 | -0.46 | 0. 96 |
| Left-Caudate | -1.53 | 0.14114 | -0.46 | 0. 96 |
| ctx-lh-rostralanteriorcingulate | 1.47 | 0.15538 | 0.34 | 0. 96 |
| Left-Pallidum | 1.4 | 0.17252 | 0.35 | 0. 96 |

**Table S6.** Top ten largest effects within the Destrieux parcellation for node degree. “Corr_p” = p value corrected for 164 comparisons.

| Region | t_stat | p | d | corr_p |
| --- | --- | --- | --- | --- |
|  |  |  |  |  |
| rh_G_subcallosal | 2.26 | 0.03008 | 0.69 | 0.97 |
| rh_S_oc_middle_and_Lunatus | 2.23 | 0.03273 | 0.61 | 0.97 |
| lh_S_orbital_med-olfact | 2.08 | 0.04457 | 0.52 | 0.97 |
| lh_piriform | 2.05 | 0.04495 | 0.61 | 0.97 |
| rh_S_circular_insula_inf | 1.88 | 0.06124 | 0.62 | 0.97 |
| rh_G_occipital_middle | 1.92 | 0.06262 | 0.5 | 0.97 |
| rh_S_orbital-H_Shaped | 1.9 | 0.06557 | 0.54 | 0.97 |
| Left-Hippocampus | 1.82 | 0.07568 | 0.53 | 0.97 |
| lh_S_central | 1.64 | 0.10983 | 0.4 | 0.97 |
| rh_S_oc_sup_and_transversal | -1.62 | 0.11248 | -0.47 | 0.97 |

**Table S7.** Top ten largest effects within the Destrieux parcellation for betweenness centrality. “Corr_p” = p value corrected for 164 comparisons.

| Region | t_stat | p | d | corr_p |
| --- | --- | --- | --- | --- |
|  |  |  |  |  |
| lh_S_orbital_med-olfact | 3.99 | 0.00066 | 0.87 | 0.11 |
| lh_S_orbital-H_Shaped | 2.45 | 0.0208 | 0.59 | 0.98 |
| lh_piriform | 2.35 | 0.0234 | 0.51 | 0.98 |
| lh_S_central | 2.26 | 0.03207 | 0.51 | 0.98 |
| rh_G_occipital_middle | 2.0 | 0.05634 | 0.49 | 0.98 |
| lh_G_oc-temp_med-Parahip | -1.93 | 0.07112 | -0.65 | 0.98 |
| Left-Hippocampus | 1.86 | 0.07366 | 0.45 | 0.98 |
| rh_S_orbital_med-olfact | 1.83 | 0.07834 | 0.46 | 0.98 |
| rh_G_front_sup | -1.74 | 0.0893 | -0.56 | 0.98 |
| rh_S_oc_middle_and_Lunatus | 1.71 | 0.08957 | 0.36 | 0.98 |

**Table S8.** Top ten largest effects within the Schaefer2018 parcellation for node degree. “Corr_p” = p value corrected for 164 comparisons.

| Region | t_stat | p_val | cohen_d | corr_p |
| --- | --- | --- | --- | --- |
|  |  |  |  |  |
| 17Networks_LH_VisCent_ExStr_4 | 3.25 | 0.00262 | 0.87 | 0.56 |
| 17Networks_LH_LimbicB_OFC_1 | 2.91 | 0.00624 | 0.71 | 0.6 |
| 17Networks_RH_LimbicB_OFC_2 | 2.79 | 0.00838 | 0.73 | 0.6 |
| 17Networks_LH_ContA_PFCl_3 | 2.52 | 0.01691 | 0.67 | 0.9 |
| Left-Hippocampus | 2.19 | 0.03442 | 0.64 | 0.96 |
| 17Networks_RH_SomMotB_S2_1 | 2.12 | 0.04079 | 0.56 | 0.96 |
| lh_piriform | 2.05 | 0.0427 | 0.52 | 0.96 |
| 17Networks_RH_DefaultB_PFCv_1 | -2.09 | 0.04411 | -0.53 | 0.96 |
| 17Networks_LH_VisCent_ExStr_2 | 2.02 | 0.05092 | 0.51 | 0.96 |
| 17Networks_LH_ContB_Temp_1 | 1.93 | 0.06148 | 0.52 | 0.96 |

**Table S9.** Top ten largest effects within the Schaefer2018 parcellation for betweenness centrality. “Corr_p” = p value corrected for 164 comparisons.

| Region | t_stat | p_val | cohen_d | corr_p |
| --- | --- | --- | --- | --- |
|  |  |  |  |  |
| 17Networks_LH_LimbicB_OFC_1 | 2.67 | 0.00158 | 0.5 | 0.28 |
| 17Networks_RH_SalVentAttnA_ParMed_1 | 3.07 | 0.00266 | 0.66 | 0.28 |
| 17Networks_LH_VisCent_ExStr_4 | 2.81 | 0.01021 | 0.64 | 0.73 |
| 17Networks_RH_LimbicB_OFC_2 | 2.38 | 0.0226 | 0.5 | 0.93 |
| 17Networks_LH_LimbicB_OFC_2 | 2.36 | 0.02619 | 0.61 | 0.93 |
| 17Networks_RH_SomMotB_S2_1 | 2.38 | 0.02959 | 0.54 | 0.93 |
| 17Networks_RH_LimbicB_OFC_1 | 2.2 | 0.0317 | 0.47 | 0.93 |
| 17Networks_RH_SomMotA_6 | 2.06 | 0.03474 | 0.42 | 0.93 |
| 17Networks_LH_DorsAttnA_ParOcc_1 | 2.16 | 0.04845 | 0.44 | 0.99 |
| 17Networks_LH_VisCent_ExStr_2 | 1.98 | 0.05151 | 0.41 | 0.99 |

**4. Whole network analysis: correlations with the duration of epilepsy**


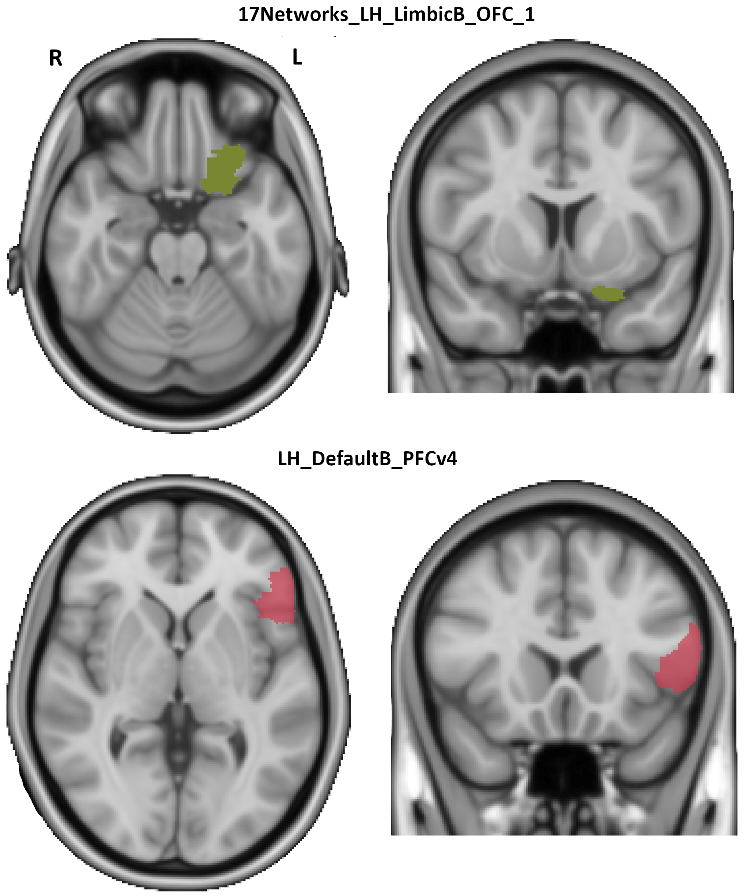


**Figure S1**. Visualization of regions with significant correlations (17Networks_LH_LimbicB_OFC_1) and anticorrelations (LH_DefaultB_PFCv4) with the duration of epilepsy from the exploratory whole-network analysis.

**Table S10.** Top ten largest effects within the Desikan parcellation for node degree. “Corr_p” = p value corrected for 82 comparisons

| Region | r_s_ | p | p_corr |
| --- | --- | --- | --- |
|  |  |  |  |
| lh_piriform | 0.39 | 0.00181 | 0.15 |
| ctx-rh-transversetemporal | 0.36 | 0.0041 | 0.17 |
| ctx-rh-frontalpole | -0.33 | 0.00883 | 0.24 |
| Left-Hippocampus | 0.31 | 0.0124 | 0.25 |
| ctx-lh-frontalpole | -0.29 | 0.022 | 0.31 |
| ctx-rh-entorhinal | -0.28 | 0.02907 | 0.31 |
| Left-Caudate | -0.27 | 0.0313 | 0.31 |
| ctx-rh-lateralorbitofrontal | 0.27 | 0.0344 | 0.31 |
| ctx-rh-parsorbitalis | -0.26 | 0.0405 | 0.31 |
| ctx-lh-bankssts | 0.26 | 0.0415 | 0.31 |

**Table S11.** Top ten largest effects within the Desikan parcellation for betweenness centrality. “Corr_p” = p value corrected for 82 comparisons

| Region | r_s_ | p | p_corr |
| --- | --- | --- | --- |
|  |  |  |  |
| Left-Hippocampus | 0.39 | 0.00137 | 0.11 |
| lh_piriform | 0.35 | 0.00469 | 0.19 |
| ctx-rh-frontalpole | -0.32 | 0.0107 | 0.29 |
| ctx-lh-parsopercularis | -0.28 | 0.02536 | 0.39 |
| ctx-rh-parsorbitalis | -0.28 | 0.02641 | 0.39 |
| Right-Amygdala | 0.27 | 0.03016 | 0.39 |
| ctx-lh-frontalpole | -0.26 | 0.0378 | 0.39 |
| ctx-lh-pericalcarine | -0.25 | 0.0501 | 0.39 |
| ctx-rh-lateralorbitofrontal | 0.25 | 0.05185 | 0.39 |
| ctx-rh-entorhinal | -0.24 | 0.0536 | 0.39 |

**Table S12.** Top ten largest effects within the Destrieux parcellation for node degree. “Corr_p” = p value corrected for 164 comparisons

| Region | r_s_ | p | p_corr |
| --- | --- | --- | --- |
|  |  |  |  |
| lh_G_front_sup | -0.42 | 0.000702 | 0.11 |
| rh_G_front_sup | -0.38 | 0.002242 | 0.18 |
| rh_G_front_inf-Triangul | -0.3 | 0.0157 | 0.47 |
| lh_piriform | 0.3 | 0.0163 | 0.47 |
| rh_G_subcallosal | 0.29 | 0.0201 | 0.47 |
| lh_S_central | 0.29 | 0.0216 | 0.47 |
| rh_Pole_occipital | -0.29 | 0.02178 | 0.47 |
| rh_G_oc-temp_med-Lingual | -0.28 | 0.0247 | 0.47 |
| Right-Caudate | -0.27 | 0.0293 | 0.47 |
| rh_G_and_S_transv_frontopol | -0.27 | 0.0304 | 0.47 |

**Table S13.** Top ten largest effects within the Destrieux parcellation for Betweenness centrality. “Corr_p” = p value corrected for 164 comparisons

| Region | r_s_ | p | p_corr |
| --- | --- | --- | --- |
|  |  |  |  |
| rh_S_orbital-H_Shaped | 0.39 | 0.00179 | 0.2 |
| rh_G_subcallosal | 0.38 | 0.00242 | 0.2 |
| lh_piriform | 0.36 | 0.0034 | 0.22 |
| lh_S_orbital-H_Shaped | 0.31 | 0.0126 | 0.42 |
| lh_S_central | 0.31 | 0.0136 | 0.42 |
| lh_Lat_Fis-post | 0.3 | 0.0164 | 0.42 |
| lh_G_front_inf-Orbital | -0.29 | 0.0215 | 0.42 |
| lh_S_circular_insula_ant | 0.29 | 0.02313 | 0.42 |
| lh_Pole_temporal | -0.27 | 0.03246 | 0.42 |
| rh_S_collat_transv_post | -0.27 | 0.0336 | 0.42 |

**Table S14.** Top ten largest effects within the Schaefer2018 parcellation for node degree. “Corr_p” = p value corrected for 214 comparisons

| Region | r_s_ | p | p_corr |
| --- | --- | --- | --- |
|  |  |  |  |
| 17Networks_LH_DefaultB_PFCv_4 | -0.45 | 0.000169 | 0.03402 |
| 17Networks_LH_LimbicB_OFC_1 | 0.44 | 0.000318 | 0.03402 |
| 17Networks_LH_VisCent_ExStr_1 | -0.31 | 0.0123 | 0.57 |
| Left-Hippocampus | 0.31 | 0.0138 | 0.57 |
| lh_piriform | 0.3 | 0.0145 | 0.57 |
| 17Networks_RH_SomMotB_S2_1 | 0.29 | 0.0183 | 0.57 |
| 17Networks_LH_ContB_Temp_1 | 0.29 | 0.0185 | 0.57 |
| 17Networks_LH_ContA_PFCl_3 | 0.28 | 0.0244 | 0.63 |
| 17Networks_RH_DefaultB_PFCv_1 | -0.28 | 0.0265 | 0.63 |
| 17Networks_RH_VisCent_ExStr_5 | -0.27 | 0.0319 | 0.68 |

**Table S15.** Top ten largest effects within the Schaefer2018 parcellation for betweenness centrality. “Corr_p” = p value corrected for 214 comparisons

| Region | r_s_ | p | p_corr |
| --- | --- | --- | --- |
|  |  |  |  |
| 17Networks_LH_LimbicB_OFC_1 | 0.5 | 0.0000341 | 0.00731 |
| 17Networks_LH_LimbicB_OFC_2 | 0.39 | 0.00167 | 0.18 |
| 17Networks_RH_ContB_PFCld_1 | -0.36 | 0.00387 | 0.28 |
| 17Networks_LH_DefaultB_PFCv_4 | -0.34 | 0.00716 | 0.38 |
| 17Networks_LH_VisCent_ExStr_1 | -0.31 | 0.0122 | 0.47 |
| lh_piriform | 0.31 | 0.0133 | 0.47 |
| 17Networks_LH_SomMotA_4 | -0.29 | 0.0229 | 0.6 |
| 17Networks_LH_LimbicA_TempPole_1 | -0.28 | 0.0245 | 0.6 |
| 17Networks_LH_DefaultC_Rsp_1 | 0.28 | 0.025 | 0.6 |
| 17Networks_RH_DefaultB_PFCv_1 | -0.27 | 0.0315 | 0.66 |

**5. Centrality of left olfactory cortex and seizure control after epilepsy surgery**

**A)** Characteristics of the sample of 28 operated patients.

| **Characteristics** | **Overall n (%)** | **ILAE I, n (%)** | **ILAE II-VI, n (%)** |
| --- | --- | --- | --- |
| **Sex** |  |  |  |
| - male | 10 (35.7) | 5 (35.7) | 5 (35.7) |
| - female | 18 (64.3) | 9 (64.3) | 9 (64.3) |
|  |  |  |  |
| **Laterality of TLE** |  |  |  |
| - left | 19 (67.9) | 7 (50) | 12 (85.7) |
| - right | 9 (32.1) | 7 (50) | 2 (14.3) |
|  |  |  |  |
| **Surgical procedure** |  |  |  |
| - SAHE | 21 (75) | 9 (64.3) | 12 (85.7) |
| - ATLR | 1 (3.6) | 1 (7.1) | - |
| - Tailored resection | 6 (21.4) | 4 (28.6) | 2 (14.3) |
|  |  |  |  |
| **Drug response** |  |  |  |
| - **resistant** | 20 (71.4) | 7 (50) | 13 (92.9) |
| - **not resistant** | 6 (21.4) | 5 (35.7) | 1 (7.1) |
| - **unclear** | 2 (7.1) | 2 (14.3) | - |
|  |  |  |  |
| **Mean post-operative FU (range) in months** | 52.7 (18, 129) | 57 (21-129) | 48.4 (18 – 83) |

**Table S16.** Characteristics of the population evaluated for seizure freedom after epilepsy surgery. FU = Follow-Up. One subject who did not become seizure free after epilepsy surgery achieved subsequent seizure freedom on ASM. Five subjects who met criteria for being labeled as drug-sensitive (cf. Methods) received epilepsy surgery and were subsequently seizure free. Note that this is no contradiction, as seizure freedom on ASM was < 1 year in these cases, so that they were not drug resistant, but also not clearly seizure free > 1 year. In two subjects, surgery followed swiftly after epilepsy diagnosis, so that a drug response could not be evaluated.

**B)** Results of permutation testing of preoperative centrality metrics in the ILAE 1 outcome group vs. patients with recurring seizures (ILAE 2-6)

| **Parcellation** | **Region** | **t-stat** | **p (uncorrected)** | **Cohen’s d** |
| --- | --- | --- | --- | --- |
| **Desikan** |  |  |  |  |
|  |  |  |  |  |
| *- degree* | lh_piriform | -1.17 | 0.26 | -0.44 |
|  |  |  |  |  |
| *- betweenness centrality* | lh_piriform | -0.35 | 0.74 | -0.13 |
|  |  |  |  |  |
| **Destrieux** |  |  |  |  |
|  |  |  |  |  |
| *- degree* | lh_piriform | -1.22 | 0.24 | -0.46 |
|  | lh_S_orbital_med-olfact | -1.02 | 0.32 | -0.39 |
|  |  |  |  |  |
| *- betweenness centrality* | lh_piriform | -0.75 | 0.49 | -0.28 |
|  | Lh_S_orbital_med_olfact | -0.14 | 0.88 | -0.05 |
|  |  |  |  |  |
| **Schaefer** **2018** |  |  |  |  |
|  |  |  |  |  |
| *- degree* | lh_piriform | -1.54 | 0.14 | -0.58 |
|  | 17Networks_LH_LimbicB_OFC_1 | -2.51 | **0.0117** | -0.95 |
|  |  |  |  |  |
| *- betweenness centrality* | lh_piriform | -1.02 | 0.43 | -0.39 |
|  | 17Networks_LH_LimbicB_OFC_1 | -1.49 | **0.0437** | -0.57 |
|  |  |  |  |  |

**Table S17.** Results from a two-tailed permutation test contrasting the centrality of left olfactory regions in patients who became seizure free (ILAE I) after epilepsy surgery vs. patients with continued seizures (ILAE II-VI). Only olfactory regions identified in the prior analysis of drug resistance were investigated in this sample of 28 patients. Uncorrected p-values < 0.05 are highlighted. Negative effects and t-values indicate greater centrality in subjects who were not seizure free after epilepsy surgery.

**6. Exemplary tractograms of the ipsilateral piriform cortex**

In this section, streamlines seeded from the ipsilateral piriform cortex are displayed for visualization of generated paths and of potential seeding of the neighboring temporal stem due to partial volume effects. This visualization was conducted using MRTRIX [1], and therefore using different algorithms for estimation of fibre orientation distribution functions (i.e., constrained spherical deconvolution, CSD, and the iFOD2 algorithm) and for fibre tracking. Descriptive visualizations were conducted using this alternative software as streamlines were not accessible from the probtrackx2 algorithm from FSL.

**Methods**

In three exemplary subjects, single-shell tissue response functions and fiber orientation density functions were estimated using CSD and the iFOD2 algorithm [2,3]. Subsequently, intensity normalization was conducted (mtnormalize) and 500000 streamlines were seeded at random from a mask of the ipsilateral piriform cortex. Fibre tracking was conducted using the tckgen algorithm with anatomically constrained tractography [4]. Here, the same brain mask was used to exclude connections via CSF or adjacent gyri as in the main manuscript. To account for overrepresentation of long projections inherent to CSD, the SIFT algorithm was applied to filter individual tractograms, resulting in 50k remaining streamlines per patient [5]. Detailed descriptive reports of individual tractograms were reported using the MRTRIX viewer mrview and images were put together using GIMP (<https://www.gimp.org/>).

**Results**

Detailed tractograms of three exemplary patients (2 left TLE, 1 right TLE, 2 drug resistant) are reported. Note that MRTRIX was used to generate streamlines, as FSL’s tractography output produces heatmaps of streamline visitation counts, which do not account for fibre orientations.

Seeding of the temporal stem due to partial volume effects was minimal (Figures S2-7). Strong interhemispheric connectivity via the anterior commissure was observed, as well as fibres fanning horizontally from the PC into the temporal stem. Meanwhile, strong connectivity to the orbitofronal cortex was detected, and figure S9 also highlights limbic connectivity via the ipsilateral cingulate. Connectivity via the fornix varied across subjects (Figure S9).


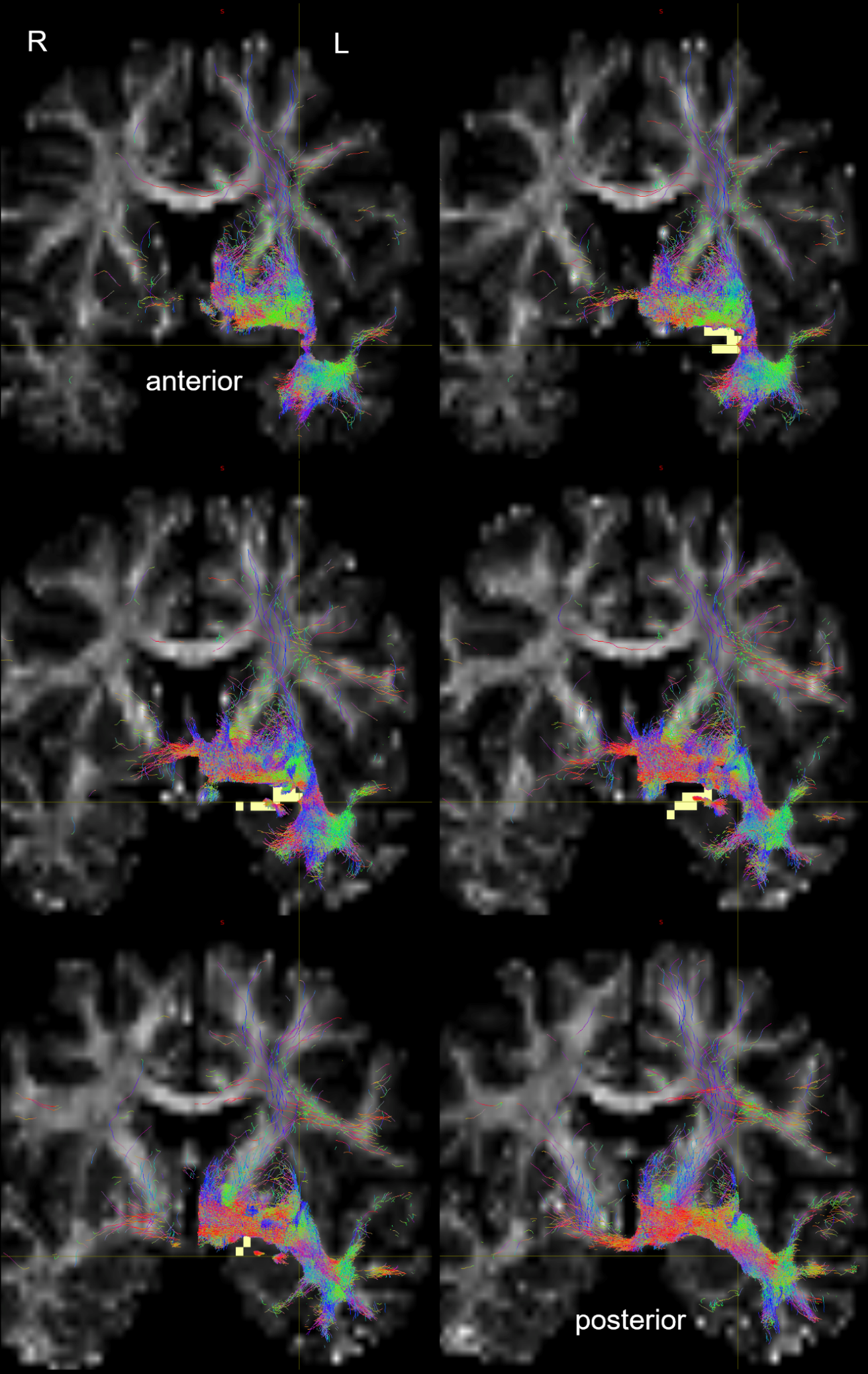


**Figure S2**. Exemplary single subject left PC (Pat. 027, left TLE, drug resistant) tractogram overlayed on the native space FA map. The ipsilateral PC mask resampled to diffusion space is shown in yellow. Slices progress from anterior to posterior from the top left to bottom right. Note strong interhemispheric connectivity via the anterior commissure. Also, note horizontal (red) tracts emerging from lateral PC voxels fanning into the temporal stem.


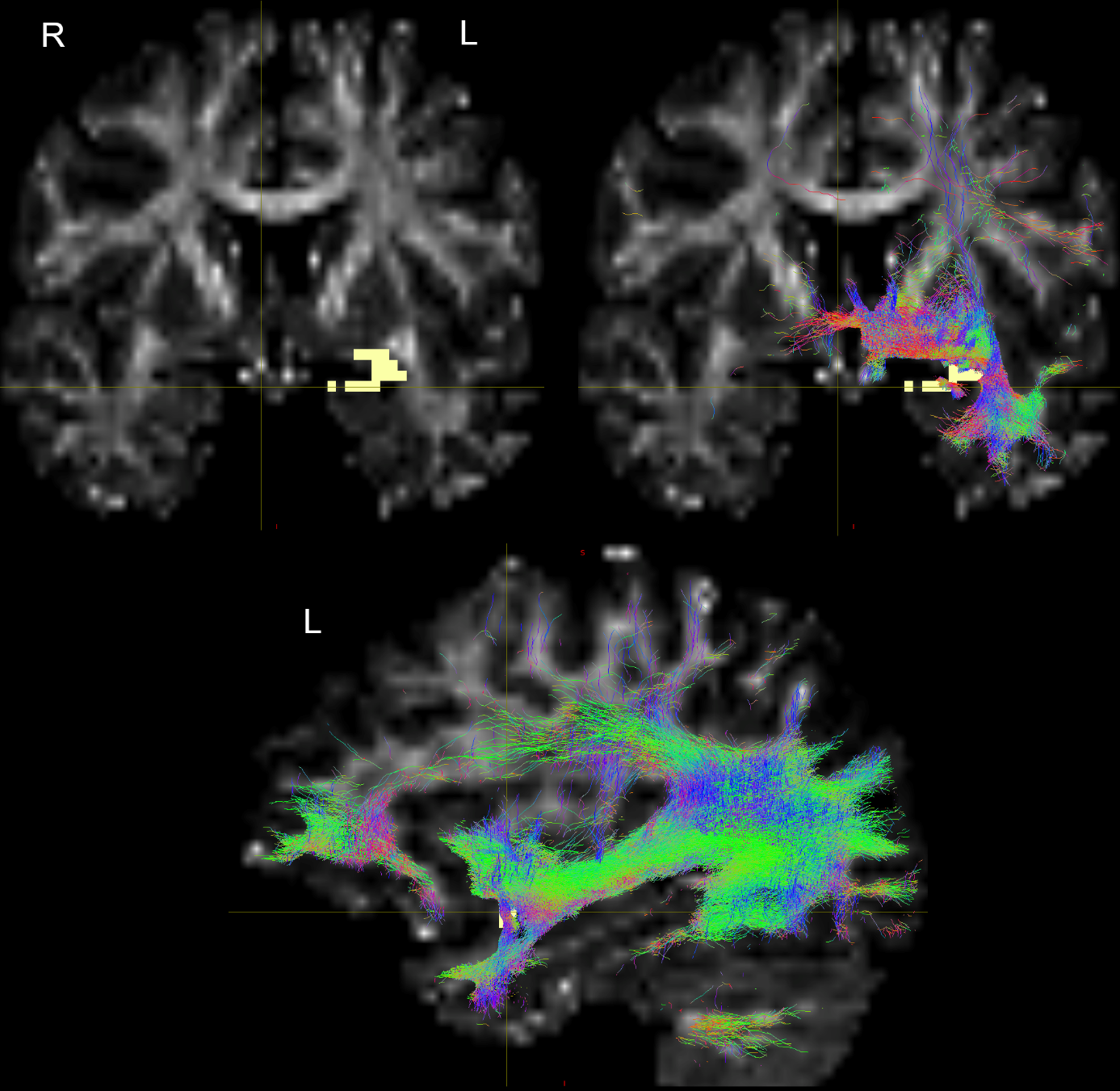


**Figure S3**. Same patient as Figure S2 (Pat. 027). The upper panels show a coronal view of the PC mask with and without the overlayed tractogram and highlight that seeding of the passing fibres of the temporal stem was minimal. The lower panel shows a sagittal view of the left hemisphere, with the crosshair centered on the lateral most voxel of the PC. Again, horizontal and oblique fibres (red and violet) are seen in this area.


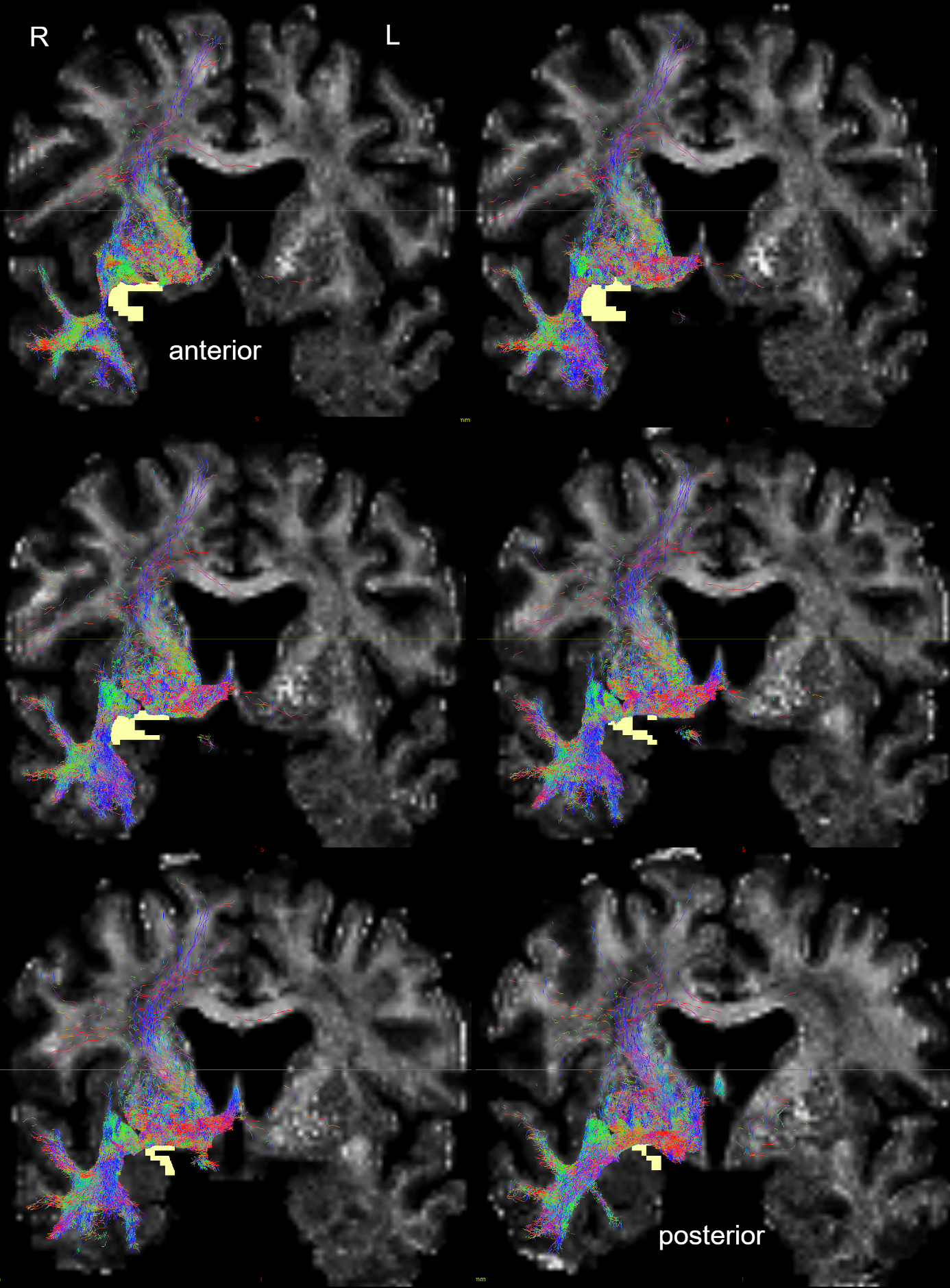


**Figure S4**. Exemplary single subject right PC (Pat. 139, right TLE, drug resistant) tractogram overlayed on the native space FA map. The ipsilateral PC mask resampled to diffusion space is shown in yellow. Slices progress from anterior to posterior from the top left to bottom right.


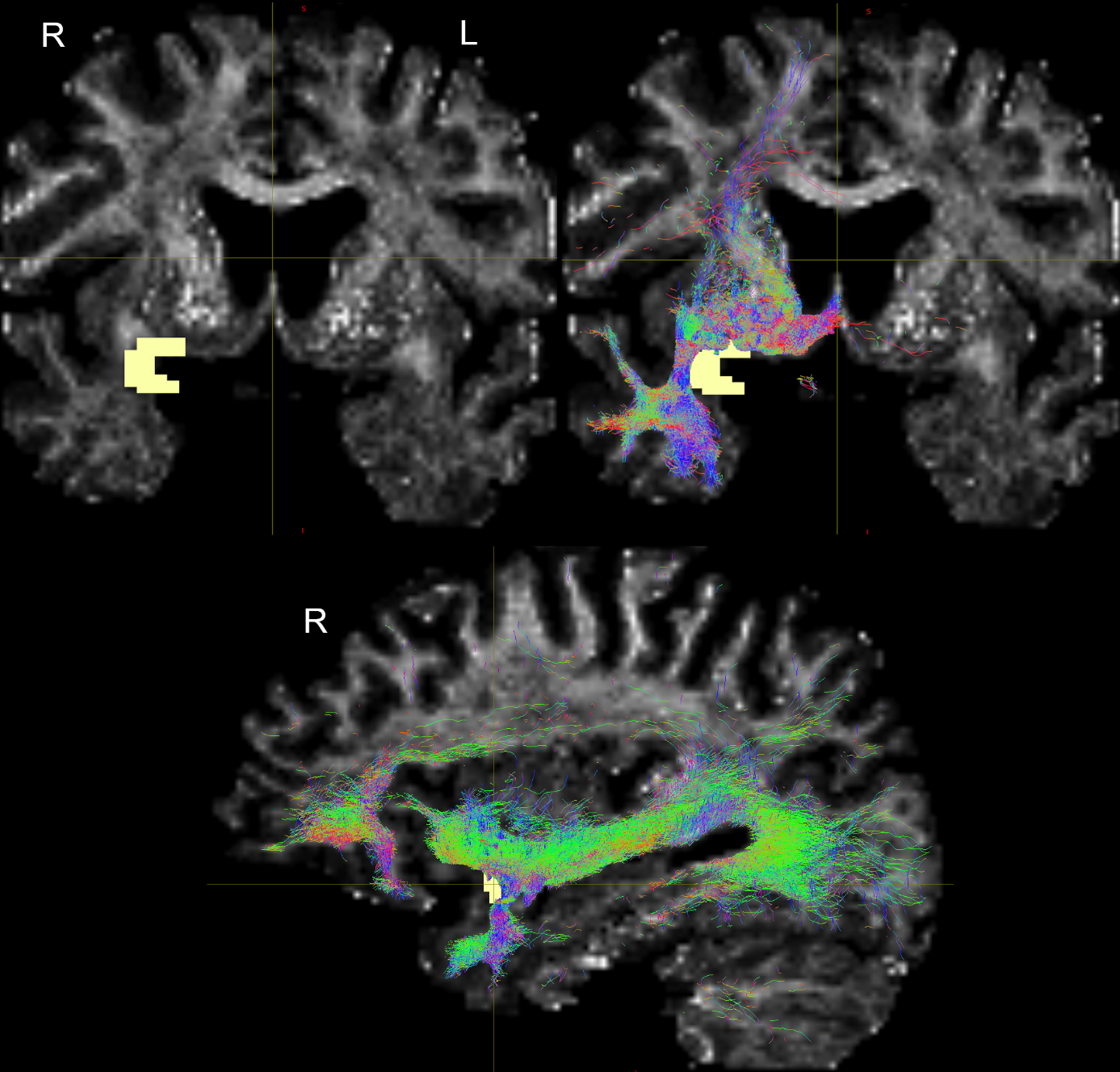


**Figure S5**. Same patient as Figure S4 (Pat. 139). The upper panels show a coronal view of the PC mask with and without the overlayed tractogram and highlight that seeding of the passing fibres of the temporal stem was minimal. The lower panel shows a sagittal view of the left hemisphere, with the crosshair centered on the lateral most voxel of the PC. Again, horizontal and oblique fibres (red and violet) are seen in dorsal parts of the PC in close vicinity to the temporal stem.


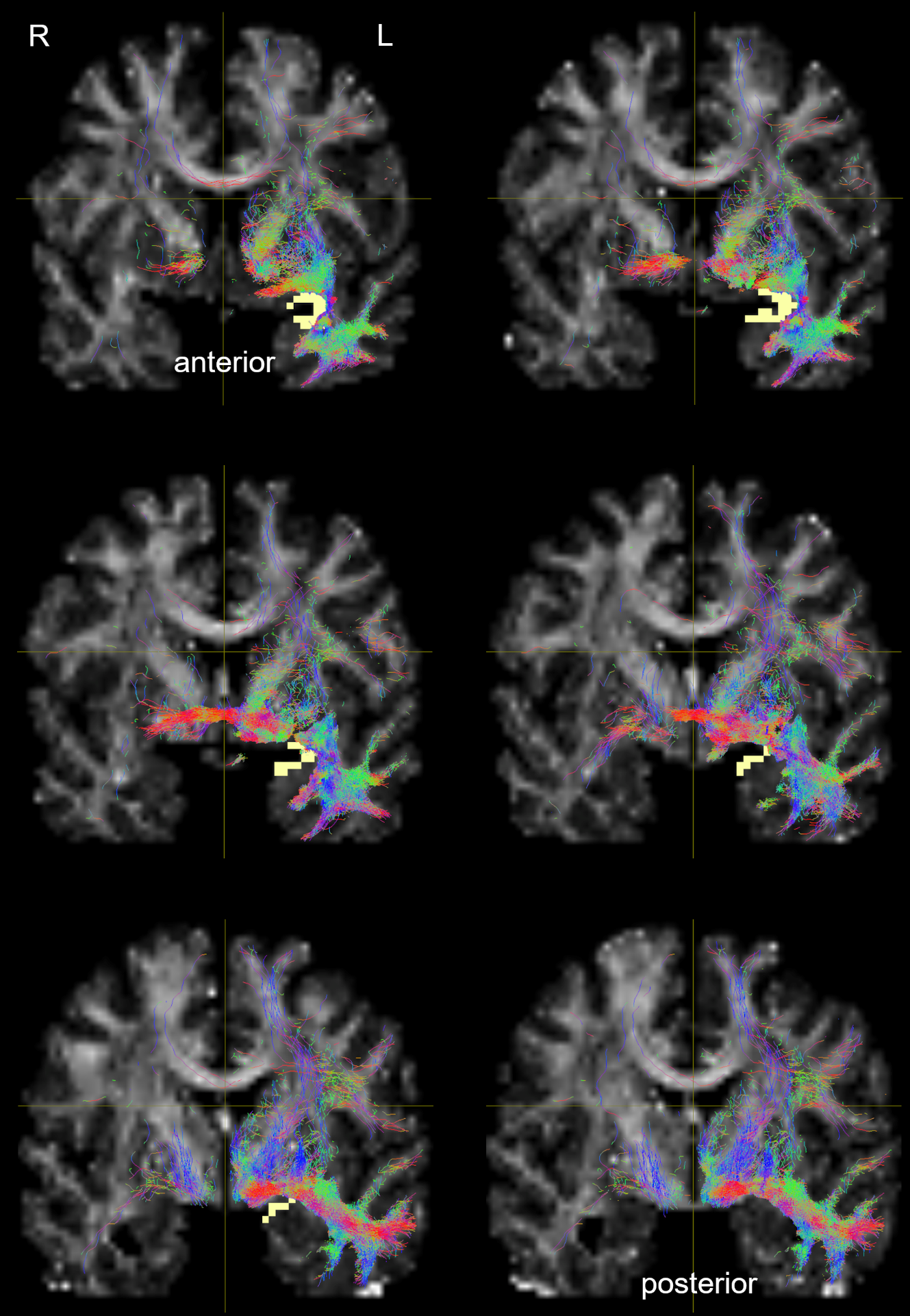


**Figure S6**. Exemplary single subject right PC (Pat. 003, left TLE, drug sensitive) tractogram overlayed on the native space FA map. The ipsilateral PC mask resampled to diffusion space is shown in yellow. Slices progress from anterior to posterior from the top left to bottom right.


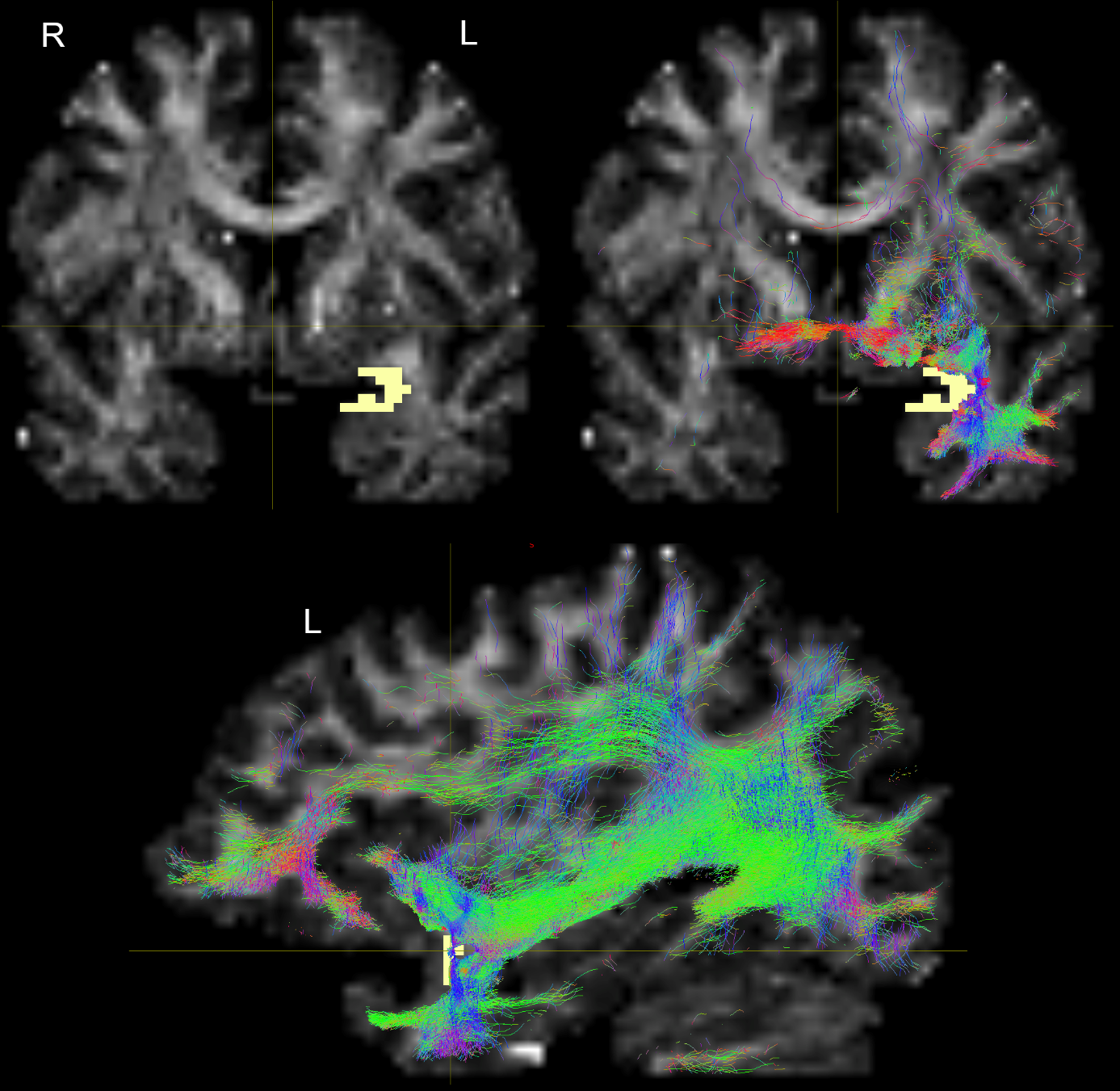


**Figure S7**. Same patient as Figure S6 (Pat. 003). The upper panels show a coronal view of the PC mask with and without the overlayed tractogram and highlight that seeding of the passing fibres of the temporal stem was minimal. The lower panel shows a sagittal view of the left hemisphere, with the crosshair centered on the lateral most voxel of the PC. Here, partial volume induced seeding of small parts of the passing temporal stem (lower panel, crosshair) can be observed, as opposed to the other two exemplary patients.

Figure S8 shows a close-up of the right PC of the second exemplary patient (Pat. 139) as well as an axial view of its connected fibres.


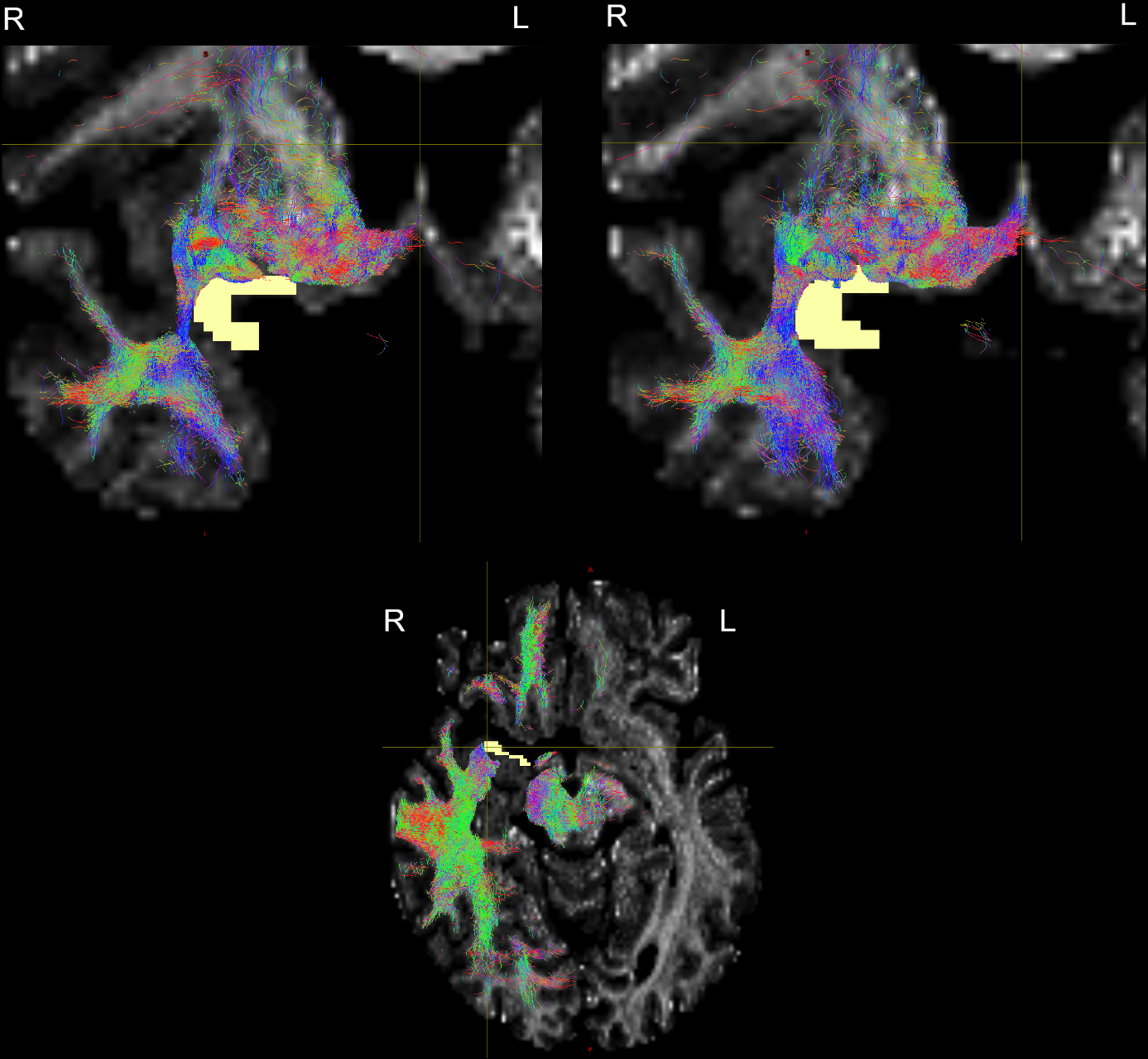


**Figure S8**. Close-up of the right PC of exemplary patient 139 (cf. Figures S4-5). Aforementioned fanning of fibres into the passing temporal stem, which is not seeded itself, can be observed. The lower panel gives an exemplary axial overview over seeded streamlines.


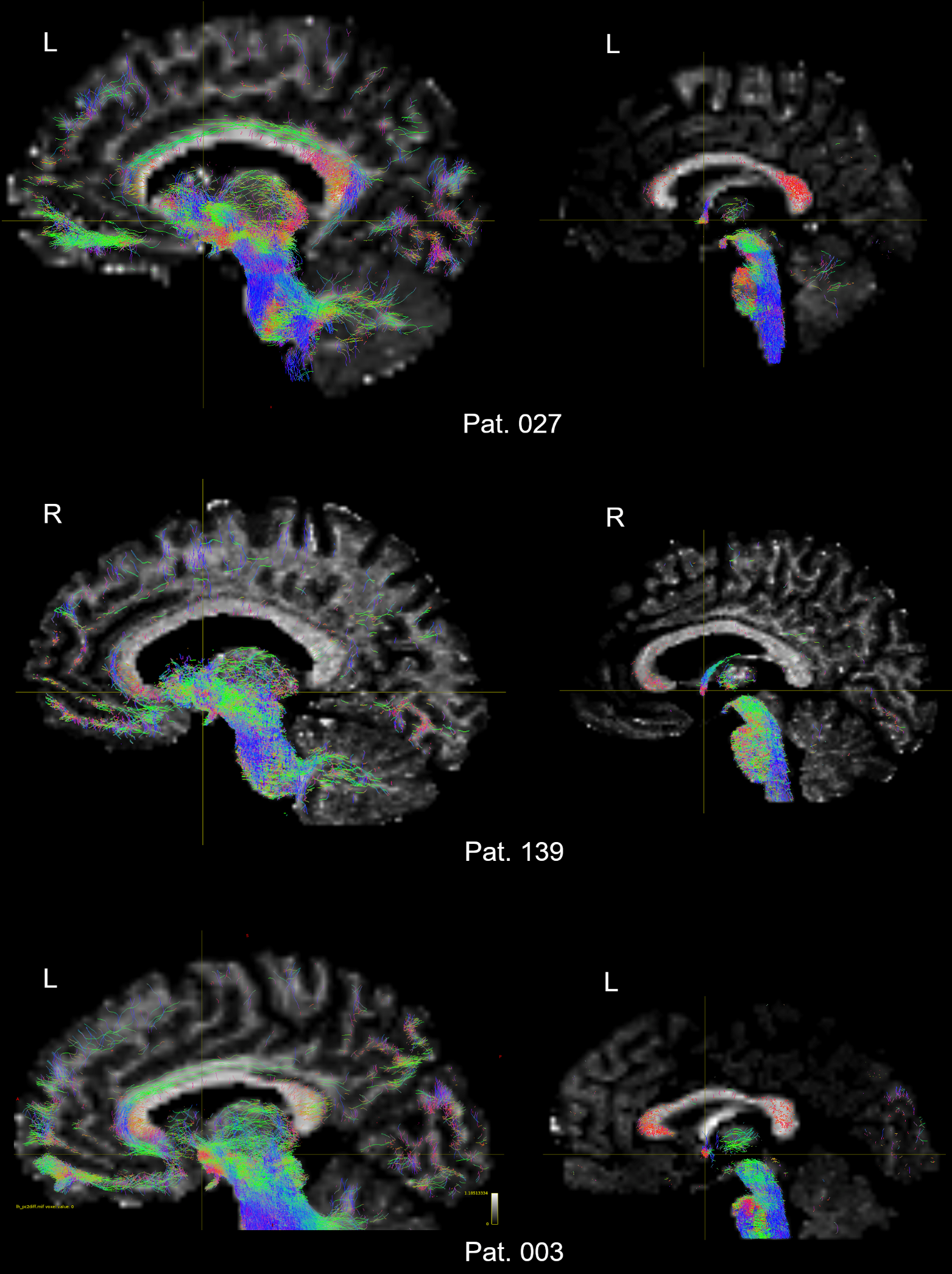


**Figure S9**. Ipsilateral connectivity with the cingulate and the fornix in three exemplary patients. The crosshair is focused on the anterior commissure. While connectivity via the cingulum can be appreciated in all 3 patients, fibres running through the fornix varied, with only sparse fibres reaching the fornix in Patient 003, for example. The background image is the individual native diffusion space FA map.

References

1. Tournier J-D**,** Smith R**,** Raffelt D*, et al.* MRtrix3: A fast, flexible and open software framework for medical image processing and visualisation. *NeuroImage* 2019; **202:** 116137

2. Tournier J-D**,** Calamante F**,** Gadian DG**,** Connelly A. Direct estimation of the fiber orientation density function from diffusion-weighted MRI data using spherical deconvolution. *NeuroImage* 2004; **23:** 1176–85

3. Tournier J-D**,** Calamante F**,** Connelly A. Robust determination of the fibre orientation distribution in diffusion MRI: non-negativity constrained super-resolved spherical deconvolution. *NeuroImage* 2007; **35:** 1459–72

4. Smith RE**,** Tournier J-D**,** Calamante F**,** Connelly A. Anatomically-constrained tractography: improved diffusion MRI streamlines tractography through effective use of anatomical information. *NeuroImage* 2012; **62:** 1924–38

5. Smith RE**,** Tournier J-D**,** Calamante F**,** Connelly A. SIFT: Spherical-deconvolution informed filtering of tractograms. *NeuroImage* 2013; **67:** 298–312
